# Internet-Based Attention Bias Modification for Social Anxiety: A Randomised Controlled Comparison of Training towards Negative and Training Towards Positive Cues

**DOI:** 10.1371/journal.pone.0071760

**Published:** 2013-09-30

**Authors:** Johanna Boettcher, Linda Leek, Lisa Matson, Emily A. Holmes, Michael Browning, Colin MacLeod, Gerhard Andersson, Per Carlbring

**Affiliations:** 1 Department of Psychology, Stockholm University, Stockholm, Sweden; 2 Freie Universitaet Berlin, Berlin, Germany; 3 Department of Psychology, Umeå University, Umeå Sweden; 4 MRC Cognition and Brain Sciences Unit, Cambridge, United Kingdom; 5 Functional MRI of the Brain Centre, University of Oxford, Oxford, United Kingdom; 6 School of Psychology, University of Western Australia, Perth, Australia; 7 Department of Behavioural Sciences and Learning, Psychology, Linköping University, Linköping, Sweden; 8 Department of Clinical Neuroscience, Psychiatry Section, Karolinska Institutet, Stockholm, Sweden; Chiba University Center for Forensic Mental Health, Japan

## Abstract

Biases in attention processes are thought to play a crucial role in the aetiology and maintenance of Social Anxiety Disorder (SAD). The goal of the present study was to examine the efficacy of a programme intended to train attention towards positive cues and a programme intended to train attention towards negative cues. In a randomised, controlled, double-blind design, the impact of these two training conditions on both selective attention and social anxiety were compared to that of a control training condition. A modified dot probe task was used, and delivered via the internet. A total of 129 individuals, diagnosed with SAD, were randomly assigned to one of these three conditions and took part in a 14-day programme with daily training/control sessions. Participants in all three groups did not on average display an attentional bias prior to the training. Critically, results on change in attention bias implied that significantly differential change in selective attention to threat was not detected in the three conditions. However, symptoms of social anxiety reduced significantly from pre- to follow-up-assessment in all three conditions (*d_within_*  = 0.63–1.24), with the procedure intended to train attention towards threat cues producing, relative to the control condition, a significantly greater reduction of social fears. There were no significant differences in social anxiety outcome between the training condition intended to induce attentional bias towards positive cues and the control condition. To our knowledge, this is the first RCT where a condition intended to induce attention bias to negative cues yielded greater emotional benefits than a control condition. Intriguingly, changes in symptoms are unlikely to be by the mechanism of change in attention processes since there was no change detected in bias per se. Implications of this finding for future research on attention bias modification in social anxiety are discussed.

**Trial Registration:**

ClinicalTrials.gov NCT01463137

## Introduction

Cognitive models of Social Anxiety Disorder (SAD) suggest that affected individuals experience social situations in ways that contribute to the maintenance of the disorder [1], [2]. Consistent with this, individuals with SAD display several specific cognitive and behavioural distortions, among which is a bias in attention processes. The cognitive models purport that, in social situations, individuals with SAD focus their attention on information that indicates social failure. This can either be internal threat cues (e.g., the perception of one's hands trembling), or external threat cues, (e.g., a negative facial expression in the social counterpart). It has been argued that this biased attention towards social threat information leads to negatively distorted evaluations of social situations, which elicit and amplify social fears [2].

A multitude of studies have assessed attention bias in SAD using different experimental approaches. Attention bias to external threat cues has mainly been investigated using the emotional Stroop paradigm [3], the dot-probe paradigm [4] and the spatial cueing task [5]. Recently, eye-tracking studies have shed some additional light on this attention bias [6]. In a review, Cisler and Koster [7] concluded that there is extensive evidence of attention bias to threat in all anxiety disorders, including SAD. The authors differentiated the components of biased attention to threat: attention bias *towards* threat (hypervigilance to threat and prolonged disengagement from threat) and attention bias *away* from threat (attentional avoidance). In SAD, both components have received at least some empirical support across different experimental designs. Anxiety-linked attention bias towards social threat cues has been demonstrated by studies applying the dot-probe paradigm. In this paradigm, two stimuli (e.g., one neutral and one social threat word) are simultaneously displayed on a screen for a certain length of time. Immediately afterwards, a probe appears in the location of one of the stimuli. Subjects are asked to respond to the probe (by pressing a button on the keyboard) as quickly as possible. Speeded responding to probes in the location of the social threat word, compared to probes in the location of neutral words, is taken to indicate biased attention towards threat. Studies applying the dot-probe paradigm vary regarding the duration of word exposure prior to probe onset (<200 ms, 500 ms, >1000 ms). Attentional vigilance to threat has been demonstrated at presentation times of less than 200 ms [8]–[10] and at 500 ms [11]–[16]. Two eye-tracking studies have also found that socially anxious individuals (initially) show greater attention to social threat cues than to neutral cues [6], [17]. Further evidence of an attention bias towards threat cues has also been provided by studies employing the emotional Stroop paradigm [18]–[21], and the spatial cueing task [22]. Fewer studies suggest that socially anxious individuals display *attentional avoidance* of social threat cues. Two studies using the dot-probe paradigm revealed an attention bias away from threat at 500 ms [8], [23] supported by two eye-tracking studies [17], [24].

It is worth mentioning that not all studies have revealed a significant attention bias. Several studies using the dot-probe paradigm have found no evidence of bias, some employing 500 ms stimulus exposures [10], [25]–[29], and some employing either shorter exposures [27], [30], or longer exposures [12], [31]. Despite the existence of these studies and studies demonstrating attentional avoidance of social threat cues, the weight of empirical evidence indicates that socially anxious individuals, in comparison to non-anxious controls, display an attention bias that favours social threat information.

Of course, the finding that social anxiety is commonly characterized by an attention bias to social threat does not permit conclusion that this attention bias functionally contributes to the emotional symptoms associated with this condition. Recently, investigators have sought to systematically manipulate biased attentional responding to threat using newly developed “training” variants of the probe task, designed with the intention of directly modifying such attentional selectivity [32]. Specifically, in these training variants, across a great many trials probes are presented more frequently in the locus of non-threatening rather than threatening stimuli, with the objective of encouraging participants to reduce their attention to threat. In two influential reports, Amir and colleagues [33] and Schmidt and colleagues [34] presented SAD patients with this dot-probe training task. In both studies, eight sessions of this training procedure produced substantial reduction in symptoms of social anxiety. Having assessed attention bias scores as an outcome measure, Amir et al. [33] were able to further demonstrate that the change in anxiety symptoms was mediated by the training-induced reduction of attention to threat. Clearly, when appraising whether the symptoms of social anxiety can be altered by the reduction of attention to threat, it is necessary to examine both the attentional and the emotional consequences of any procedure. Several further studies now have been conducted on participants with SAD that build on the foundational work of Amir et al. [33] and Schmidt et al. [34]. The general pattern of findings has been that when the intended bias modification procedure successfully reduces attention to threat, then it also attenuates anxiety symptoms, but such reduction of attention bias is not always forthcoming.

In a recent trial, Heeren et al. [35] found that a probe-task configured with the intention of reducing attention to threat cues successfully induced such attentional change. Heeren et al. also observed that this attenuation of attention to threat was accompanied by a reduction in behavioural and physiological measures of social anxiety. Likewise, two further studies that have successfully changed attention bias also have demonstrated that this attention change is accompanied by a positive effect on social anxiety [36], [37]. In contrast, studies that have failed to successfully induce the intended attentional change using this type of probe procedure consistently have also failed to observe any beneficial impact on social anxiety. Thus, for example, Julian, Beard, Schmidt, Powers, and Smits [29] found that a probe-task configured with the intention of reducing attention to threat did not do so to a significantly greater degree than a control condition, and neither was it more effective than the control condition in reducing social fears. Failure to reduce attention to threat has been especially common when attention training programmes have been delivered across the internet. Using internet delivery of such tasks, Boettcher, Berger, and Renneberg [28], Carlbring and colleagues [38], as well as Neubauer and colleagues [39], all have failed to document that their intended attentional training led to greater attentional change than a control condition. And, in the absence of such differential change, each of these studies also observed no differential impact of these conditions on anxiety symptoms.

So far, the reasons why attention bias modification (ABM) studies have varied in terms of their capacity to attenuate attention bias to threat and to reduce social anxiety, have not been clearly established. Meta-analyses on attention modification programmes have reported a fairly large effect of these ABM procedures on attention bias, and small to moderate effects on anxiety symptoms [40], [41]. They also identified some training-specific task characteristics that may affect the magnitude of their attentional impact. For example, ABM procedures that have delivered multiple sessions, presented stimulus pairs in a top-bottom orientation rather than a right-left orientation, and used word stimuli rather than face stimuli have yielded better results [40], [41]. This last finding is surprising, as the use of face stimuli has been promoted due to its ecological validity. Amir, Taylor, and Donohue [42] investigated potential predictors and moderators in ABM for SAD. The authors examined socio-demographic and clinical characteristics as well as cognitive disturbance measures. The only significant moderator was pre-training attention bias. Only individuals with an initially pronounced attention bias towards threat benefitted emotionally from the training procedure intended to reduce attention to threat. Amir et al. [42] did not examine whether this reflected the greater attentional impact of this ABM procedure in such participants, as plausibly might be the case. Adopting the assumption that the benefits of such training would be greatest in those displaying an initial preference for threat, and noting that about half the patients in clinical samples do not display such an attention bias, Eldar et al. [43] excluded all children who did not display an attention bias towards threat at pre-assessment from their sample when examining the impact of an effective ABM regime in childhood anxiety.

Hence, it could be hypothesized that there are differing subgroups of socially anxious individuals, some of whom display attentional vigilance for threat cues whereas others display attentional avoidance and still others might not show any attention bias at all [44]. If so, then training programmes intended to reduce attention bias towards threat may work only for the subset of anxious individuals with an attention bias towards threat. In contrast, participants whose anxiety is marked by attentional avoidance of threat may benefit instead from modification procedures designed to increase attention to threat. However, attention bias towards and attention bias away from threat are not necessarily mutually exclusive when considered within the hypervigilance-avoidance-framework [45]. The hypervigilance-avoidance theory assumes that anxious individuals initially show quick engagement with threat cues but subsequently withdraw their attention and avoid these same threat cues. As a consequence, the length of stimulus presentation in ABM procedures may be crucial in successfully training the attention bias. Unfortunately, there is yet no consensus in the literature as to specific time periods for hypervigilant and avoidant processing stages [7].

Given that the specific mechanisms that constitute attention bias in SAD are yet to be clearly established, and the role of individual differences and differences between processing stages has yet to be fully understood, an exclusive focus on attention bias towards threat may be premature. Only two studies so far have evaluated a programme training attention towards threatening cues in clinical or analogue samples. In an analogue sample, Klumpp and Amir [46] compared an ‘attend to threat’ condition with an ‘attend to neutral’ and a control condition. They found that participants of both training groups were less anxious in a subsequent speech task than participants in the control group. Heeren et al. [35] compared an ‘attend to threat’ condition with an ‘attend to positive’ condition and a control condition in individuals with SAD. The authors were able to show that the ‘attend to positive’ procedure modified attentional selectivity as intended. In contrast to Klumpp and Amir's findings, however, these authors reported that, at post-assessment, the participants of the ‘attend to threat’ condition showed more social anxiety on self-report, physiological, and behavioural measures than did participants in the ‘attend to positive’ condition or in the control condition.

The first aim of the current trial was to examine the efficacy of two attention training conditions, intended to increase attention to either threatening or positive cues, to change attentional processes and lead to improvements of social anxiety. The second aim was to examine whether the training task itself might be more effective when presenting just word stimuli or a combination of word and face stimuli. We were particularly interested in the combination of word and face stimuli as it seems the material set with the highest ecological validity. It was compared to a words only set as this had proved best in meta-analysis [40], [41]. A randomized controlled design was used in which participants were randomly allocated to receive one of three attention training interventions configured with the intention to; 1) increase attention towards threatening stimuli, 2) increase attention towards positive stimuli, or 3) not to alter attention. Additionally, within each of these groups, participants were randomized to complete training tasks in which only words or both words and faces were presented. We aimed to answer the following research questions. 1) Do either or both of the two active conditions reduce social anxiety more than does the control condition? 2) Do the two active conditions, compared to the control condition, produce the intended differential change in attentional selectivity? 3) Is the training with words alone superior to the training with words and faces? 4) Does the pre-training bias score qualify as a moderator variable?

## Methods

### Participants

The protocol for this trial and supporting CONSORT checklist are available as supporting information; see Checklist S1 and Protocol S1. The randomised controlled trial was registered at ClinicalTrials.gov (NCT01463137). The study protocol was approved by the regional ethics committee of Umeå University. Participants were recruited via advertisement in regional newspapers. Recruitment started in September 2011. Treatments were delivered in October 2011 and the follow-up-assessment was conducted in March 2012. The study was home-based with participants completing both assessment and training sessions online. After registering with their e-mail address, participants obtained detailed information on the study and were asked to give written informed consent. Participants were advised that the study aimed at modifying biased attention processes typical for individuals with social anxiety and that attention bias modification programmes had proven effective in previous scientific studies. Participants did not receive any kind of monetary compensation for participation in the study.

The selection of the participants followed two steps. First, participants were asked to fill out the outcome questionnaires and to take part in the first attention bias assessment. These included the Social Phobia Screening Questionnaire (SPSQ) [47], the Montgomery Åsberg Depression Rating Scale – Self-report version [48], and additional questions regarding current and past treatment. In a second step, participants who indicated a diagnosis of SAD according to the SPSQ were invited to take part in a telephone-administered diagnostic interview. Two advanced MSc clinical psychology students conducted the social anxiety and depression section of the Structured Clinical Interview for DSM-IV Axis I Disorders (SCID-I) [49]. All interviewers had received training in using the SCID-I.

We applied the following inclusion criteria: (a) being at least 18 years old, (b) having access to the internet, (c) meeting diagnostic criteria for a primary diagnosis of SAD (d) error rate of below 20% in the first attention bias assessment, (e) not participating in any other psychological treatment for the duration of the study, and (f) if on prescribed medication for anxiety/depression, dosage had to be constant for 3 months prior to the start of the treatment. Participants with suicidal thoughts, defined as scoring 4 or higher on item 9 of the MADRS-S, were interviewed by phone using the SAD PERSONS interview [50] to evaluate their suicidal risk. Participants indicating suicidal ideation were excluded from the study and were referred to local psychiatrists or psychologists.

**Figure 1:**
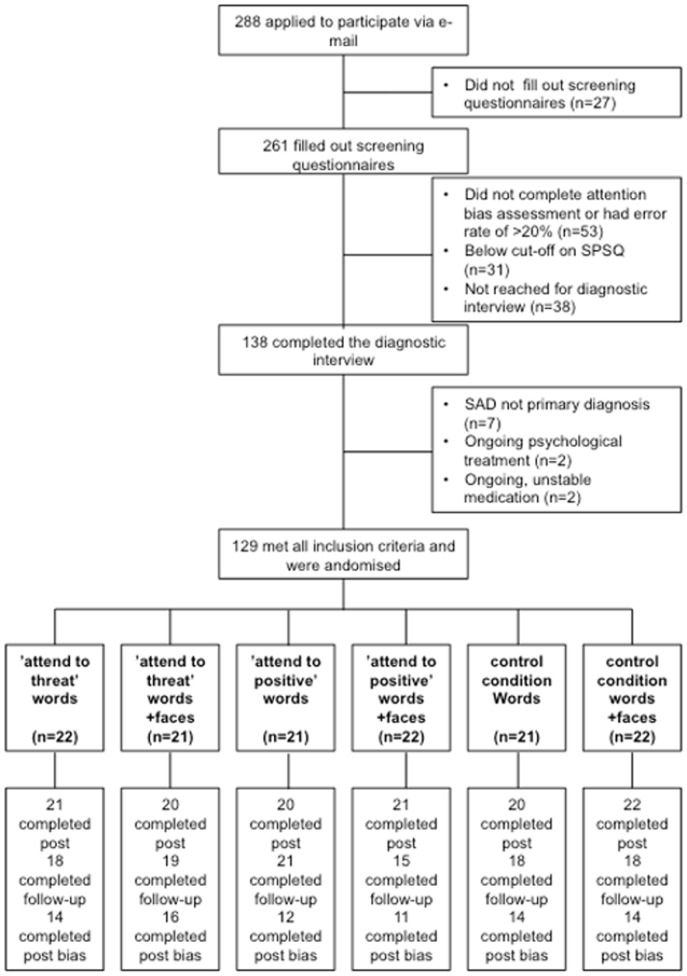
Flow chart of participants.

A total of 129 participants met all inclusion criteria and were randomised to one of the six groups (see flow chart in Figure 1). Six participants (4.7%) did not complete self-report measures at post-training and 20 participants (15.5%) failed to fill out self-report measures at follow-up-assessment. Participants were asked to complete the attention bias assessment at post-treatment in a separate email after they had filled in the self-report measures and forty-eight participants (37.2%) did not provide attention bias data. Drop-out rates did not differ between the groups (all *χ^2^* (2) <2.6, all *p*>.31).

Table 1 displays socio-demographic characteristics as well as pre-training scores of the outcome measures for the total sample and the six groups. There were no significant group differences at pre-training on any demographic variable or outcome measure.

**Table 1:** Participants' characteristics at pre-assessment in each of the six groups.

	Total (*N* = 129)		Control words (*N* = 21)		Control words + faces (*N* = 22)		Towards negative words (*N* = 22)		Towards negative words + faces (*N* = 21)		Towards positive words (*N* = 21)		Towards positive words + faces (*N* = 22)		
	*N*	*%*	*N*	*%*	*N*	*%*	*N*	*%*	*N*	*%*	*N*	*%*	*N*	*%*	Test statistics
Male	47	36%	11	52%	9	41%	4	18%	6	29%	9	43%	8	36%	*χ^2^*(5) = 6.60 *p* = .26
In relationship	64	51%	15	71%	13	59%	10	45%	8	38%	10	48%	8	41%	*χ^2^*(5) = 7.63 *p* = .18
Single	64	50%	6	29%	9	41%	12	55%	13	62%	11	52%	13	59%	
High school	5	4%	3	14%	0	0%	2	9%	0	0%	0	0%	0	0%	*χ^2^*(5) = 13.22 *p* = .20
College	34	26%	4	19%	8	36%	4	18%	6	29%	5	24%	7	32%	
University	90	70%	14	67%	14	64%	16	73%	15	71%	16	76%	15	68%	
	*Mean*	*SD*	*Mean*	*SD*	*Mean*	*SD*	*Mean*	*SD*	*Mean*	*SD*	*Mean*	*SD*	*Mean*	*SD*	
Age	38.26	12.32	42.81	11.50	39.32	11.47	35.50	13.24	35.57	9.51	38.05	13.50	38.36	13.99	*F*(5,123) = 1.03 *p* = .40
LSAS-SR	74.60	22.06	71.81	18.77	74.45	21.91	73.64	22.44	78.48	21.97	72.90	23.20	76.32	25.38	*F*(5,123) = .25 *p* = .94
SPS	37.99	13.69	37.33	14.63	37.05	14.11	36.59	15.94	40.00	11.45	39.57	12.58	37.55	14.16	*F*(5,123) = .22 *p* = .95
SIAS	50.82	13.32	48.19	13.46	47.05	13.85	51.36	13.25	55.48	11.40	54.19	12.87	48.91	14.18	*F*(5,123) = 1.4 *p* = .22
MADRS-S	15.09	7.46	15.71	8.47	16.05	7.71	14.09	7.67	15.29	6.96	13.90	7.68	15.45	6.79	*F*(5,123) = .29 *p* = .92
QOLI	0.82	1.71	0.71	1.50	1.09	1.82	1.03	1.79	0.64	1.70	0.67	1.90	0.73	1.67	*F*(5,123) = .28 *p* = .93
BAI	18.38	8.61	18.81	9.85	20.14	8.88	16.68	8.09	17.48	6.47	19.62	7.72	17.59	10.44	*F*(5,123) = .53 *p* = .76

LSAS-SR: Liebowitz Social Anxiety Scale- Self Report Version, SPS: Social Phobia Scale, SIAS: Social Interaction Anxiety Scale, MADRS-S: Montgomery Åsberg Depression Rating Scale – Self-report version, QOLI: Quality of Life Inventory, BAI: Beck Anxiety Inventory.

### Procedure

After pre-assessment, participants were randomly allocated to one of the six groups by an online true random-number service independent of the investigators. Participants and investigators remained blind to the allocation schedule until after the post-assessment. After randomisation, participants received access to a website where the attention training and control tasks were presented. Participants were asked to complete their assigned task daily for 14 days [51]. Primary and secondary outcome measures were administered over the internet prior to the training, immediately after the training (at the end of week 2) and at four months follow-up. In addition, we assessed attention bias before and after the training.

### Intervention

The training/control tasks were based on the dot-probe paradigm. Tasks were identical in all groups except for the kind of stimuli (words alone vs. words and faces) and the location of the probes. Each training/control session comprised 192 trials. In the first 96 trials of each session, stimuli were presented for 1000 ms, in the second 96 trials stimuli were presented for 500 ms. We chose to include two presentation times to take into account biased attention at varying processing stages (see Introduction). Each trial started with a 500 ms inter-trial pause consisting of a blank white screen, followed by a black fixation cross (“+”) presented in the centre of the screen for 500 ms (Arial size 14). Following the fixation cross, a pair of stimuli appeared for 500 ms or 1000 ms either consisting of two words (Arial size 16) with different emotional valence, or of two portrait images of the same person's face expressing two different facial expressions (200 pixels high; width 131, 133 or 148 pixels depending on stimulus set). Stimuli were presented in the centre of the screen one above the other in random order. On one third of the trials in each session stimulus pair members were neutral-negative, on one third they were positive-negative, and on one third they were neutral-positive. In the words only conditions, stimuli consisted of 111 neutral words, 111 social threat words, and 111 positive words [52]. In the words and faces conditions, words were combined with images of 62 men and 62 women displaying positive (happiness), neutral, or negative (disgust) facial expressions. Images were derived from three different data sets: the Umeå University Database of Facial Expressions [53] the Karolinska Directed Emotional Faces [54] and the Matsumoto and Ekman's Japanese and Caucasian Facial Expressions of Emotion [55].

After either 500 ms or 1000 ms exposure, the pair of stimuli was replaced with a probe, which appeared in the position of either the upper or the lower previously displayed stimulus. The probe presented was either a ‘less-than sign’ (“<”) (Arial size 16) or a ‘greater-than sign’ (“>”). Participants were instructed to respond as quickly and accurately as possible to the probe by pressing the corresponding button on the keyboard. The probe remained on the screen until a response was given, after which it disappeared, and the next trial began.

The condition intended to increase attention to threat cues (which will be referred to as the ‘attend to threat’ condition), and the condition intended to increase attention to positive cues (which will be referred to as the ‘attend to positive’ condition), and the control condition, differed only in the frequency with which the probes replaced neutral, positive, and negative stimuli. In the ‘attend to threat’ condition the probe always replaced the more negative stimuli which was intended to establish a link between the more negative cue position and the probe position. Therefore, on neutral-negative trials, the probe would appear in the location of the negative word or face, and on neutral-positive trials, the probe would appear in the location of the neutral stimulus. In the ‘attend to positive’ condition, the probe instead always would appear in the location of the more positive stimulus. In the control condition, no contingency between the position of the differing valenced stimuli and probe position was established, and the probe appeared with equal frequency in the location of the more negative and the more positive stimuli. In order to simulate the emotional arousal thought to be associated with coming in person to the laboratory for training sessions, we asked participants before each session to provoke anxiety by conducting specified exercises, such as placing an anxiety provoking phone call. Participants were advised that the stimulation of emotional arousal would likely increase the efficacy of the training tasks and that they should aim at provoking a moderate level of anxiety. No further instruction was provided.

### Assessment and outcome measures

#### Attention bias

The participants' attention bias was assessed prior to and after the 14-day training period. The attention bias assessment employed the same dot-probe tasks used in the training and presented stimuli for 500 ms. The assessment consisted of 96 trials in which participants were presented with negative, neutral, and positive words and faces. As in the training sessions, assessment trials were balanced regarding the position of the stimuli on the computer screen, but probes appeared equally often in the locations of negative, neutral, and positive stimuli. The attention bias assessment produced response times for every participant to probes appearing in the location of the more negative or the more positive cues across the three types of trials (negative-positive, negative-neutral, neutral-positive). For each participant, we calculated the mean response time to probes in each position, on each type of trial, eliminating response latencies for inaccurate trials (1.5% of all trials), those less than 200 ms or greater than 2000 ms (0.6% of all trials), and those that differed more than two standard deviations from an individual's mean (3.4% of all trials). We calculated an attention bias to threat index from the remaining assessment trials by subtracting mean reaction times to the relatively more negative cues from mean reaction times to the relatively more positive cues (in negative-positive trials: RT (positive)-RT (negative); in negative-neutral trials: RT (neutral)-RT (negative), in positive-neutral trials: RT (positive)-RT (neutral)). A positive attention bias to threat index reflects an attention bias towards threat and away from positive cues. A negative attention bias to threat index indicates an attention bias away from threat cues and towards positive cues.

#### Emotional measures

We administered the following social anxiety scales as primary emotional outcome measures of the study: the self-report version of the Liebowitz Social Anxiety Scale (LSAS-SR) [56], the Social Phobia Scale, and the Social Interaction Anxiety Scale (SPS & SIAS) [57]. In addition, as secondary outcome measures, we administered the self-rated version of the MADRS [48] to assess depressive symptoms, the Beck Anxiety Inventory to assess general anxiety (BAI) [58], and the Quality of Life Inventory (QOLI) [59]. All questionnaires were administered online. Adequate psychometric properties have previously been demonstrated for internet-administered questionnaires relating to SAD [60]–[62]. We also asked participants how often they had conducted fear-provoking exercises prior to the training sessions. Answers ranged between 0% (never) to 100% (before each session).

### Statistical Analyses

All analyses on change in attention bias, on change in social anxiety, and on secondary outcome measures were conducted as intention-to-treat analyses using a mixed model approach. Linear mixed models, which are also sometimes referred to as multilevel linear models, mixed effects models or hierarchical linear models, can account for non-independence in the data as it usually occurs within repeated measurements over time. To account for non-independence in the data, we applied autoregressive covariance structures for all analyses including more than two assessment points. Linear mixed models also estimate missing data, obviating the need for last observation carried forward or other missing data methods and are therefore appropriate to analyse repeated measures data with dropouts [63], [64].

In a first step, we calculated a social anxiety composite score. Following the procedures recommended by Rosnow and Rosenthal [65] and applied by Clark et al. [66], the social anxiety composite score was generated by converting each social phobia scale (LSAS-SR, SIAS, SPS) across all assessment points to z-scores, and then by averaging across the measures. The social anxiety composite was then entered as dependent variable in the mixed models in order to examine change in social anxiety in the three training conditions. Planned contrasts analysed the differential change in social anxiety from pre- to post- and from pre- to follow-up-assessment in a) the training towards threat compared to the control group and b) the training towards positive compared to the control group.

In a second step, we analysed change in attention bias in the different conditions. The attention bias to threat index at pre- and post-assessment was entered as dependent variable in the mixed models. Non-orthogonal contrasts were conducted to compare a) the training towards negative group with the control group and b) the training towards positive group with the control group.

Mixed models were carried out in R Version 2.15 [67] and were fitted with nlme [68]. In this approach, main and interaction effects are evaluated on the basis of their contribution to an increase of goodness of model fit [64]. As in repeated measures ANOVA, three effects were evaluated. First, the main effect of time was estimated by comparing the goodness of fit of a model that included the factor ‘time’ to a baseline model, which only included the participants as random factor. In this approach, a significant main effect of time is associated with a significant increase of model fit. The increase of model fit is *χ^2^* -distributed. Second, the main effect of ‘treatment condition’ was evaluated by comparing the fit of a model that additionally included the factor ‘treatment condition’ to the previous model that included only the main effect of time. Third, the interaction effect of ‘time × training condition’ was evaluated by comparing the goodness of fit of a model that included the interaction of time × training condition to the goodness of fit of the previous model that only included the two main effects. Planned comparisons were conducted within this most complex model including both main effects and the interaction of time treatment × condition.

For all analyses on primary and secondary outcomes, we calculated within and between group effect sizes based on Cohen's formula using pooled standard deviations (M_1_–M_2_)/SD_pooled_) for the completer sample.

To investigate potential predictors and moderators of change in social anxiety and attention bias, we included variables of interest as additional factors in the mixed model analyses. The first variable of interest was the stimulus condition (words versus words and faces). The second variable was the amount of fear provoking exercises participants reported completing prior to the training/control sessions and the third variable of interest was the pre-training attention bias to threat index. Each of these variables was entered as an independent factor in two mixed model analysis. The first mixed model included the social anxiety scores as dependent variable, the second the attention bias to threat index. Both models also included the factors ‘time’, ‘treatment condition’, and ‘time × treatment condition’. In this approach, a variable of interest is to be classified as a predictor of change if it has a significant influence on change in the dependent variable (significant increase of model fit when adding the interaction of ‘variable of interest × time’). A variable of interest is to be considered as a moderator of change if the model fit increases significantly when adding the interaction term of ‘training condition × variable of interest × time’ [69].

Clinical significant change at post- and follow-up assessment was determined for the completer sample and based on the LSAS-SR as this scale encompasses both fear and avoidance of performance and interaction situations. In a first step, reliable change according to the Reliable Change Index [70] was determined by using the retest reliability of *r_tt_* = .83 reported by Baker et al. [56]. In a second step, a cut-off score was calculated for the formula ‘c’ reported by Jacobson and Truax [70] and based on normative data by Fresco et al. [71]. Based on these assumptions, clinically significant improvement for a given participant was defined as showing a pre-post/pre-follow-up change score of 25 or greater and a post/follow-up test score below 43.3.

## Results

Participants were asked to complete the probe task daily for 14 days. On average, participants adhered well to the training and carried out this task 11.36 times (SD = 4.25) in two weeks. Groups did not differ in terms of the frequency with which they completed the task (*F*(5,123)  = 1.74, *p* = .13). After this 14 day period, participants were asked how satisfied they were with the training they had received. Answers ranged between 1 ( =  very dissatisfied) to 4 ( =  very satisfied). On average, participants were moderately satisfied (Mean  = 2.70, SD = .72) with no differences in satisfaction between the groups (*F*(5,117)  = 1.45, *p* = .21). The instruction to conduct fear-provoking exercises prior to each training session was followed, on average, prior to only 27.4% (SD = 25.73) of the sessions. Groups did not differ in the frequency with which they complied with this instruction (*F*(5,123)  = 0.56, *p* = .75).

### Change in social anxiety

**Figure 2:**
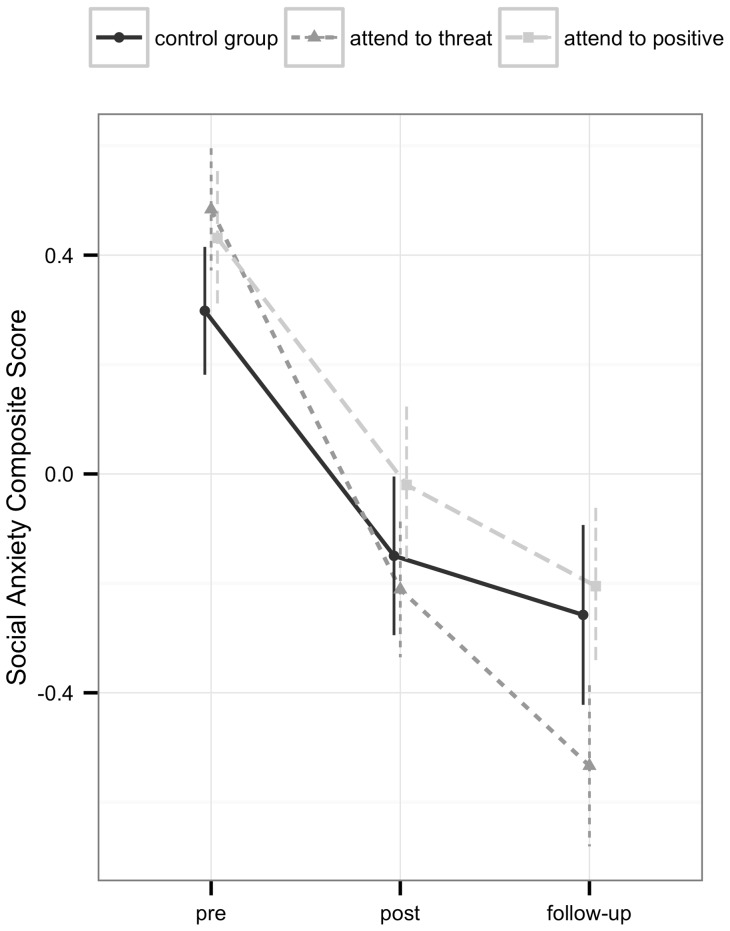
Change in social anxiety from pre- to follow-up-assessment.

Table 2 displays means, standard deviations as well as within and between group effect sizes for all social anxiety measures in the three training conditions. The mixed model analysis using the social anxiety composite score as dependent variable revealed that, across training conditions, participants improved significantly from pre- to follow-up-assessment (time: *χ^2^*(2)  = 135.11, *p*<.001). Within group effect sizes indicated moderate to large improvements (*d* = 0.52–1.24). Collapsed across all three assessment points, participants assigned to the three different training conditions did not differ significantly in their level of social anxiety (training condition: *χ^2^*(2)  = 0.79, *p* = .68). However, the rate of change in social anxiety from pre- to follow-up was not equivalent for participants who received the three training conditions (training condition × time: *χ^2^*(4)  = 11.21, *p* = .02). Figure 2 illustrates the nature of this interaction effect, showing change in the social anxiety composite score from pre- to follow-up-assessment in the three groups. Planned contrasts revealed that, compared to the control group, participants who received the ‘attend to threat’ training improved significantly more from pre- to post-assessment (*t*(226)  = −1.97, *p* = .049) and from pre-to follow-up-assessment (*t*(226)  = −12.95, *p* = .003). Between group effect sizes at post- and follow-up-assessment were small (*d* = 0.07–0.29). Participants who received the ‘attend to positive’ condition did not show any differential change in social anxiety compared to the control group, neither from pre- to post-assessment, nor from pre- to follow-up assessment (all *t*<−0.34, all *p*>.73). To follow-up the differences in means between the ‘attend to threat’ and the ‘attend to positive’ condition displayed in Figure 2, we run additional post hoc analyses that revealed that the ‘attend to threat’ group changed significantly more from pre- to follow-up-assessment than the ‘attend to positive’ group (*t*(226)  = 2.63, *p* = .01). Pre-post change rates were not significantly different between these two groups (*t*(226)  = 1.79, *p* = .07).

**Table 2:** Observed means, standard deviations, and effect sizes (Coheńs) d of primary and secondary outcome.

		*Control group*			*Attend to threat*			*Attend to positive*				
		*Mean*	*SD*	*d within*	*Mean*	*SD*	*d within*	*Mean*	*SD*	*d within*	*d between control-neg*	*d between control-pos*
Attention bias to threat index	Pre	−1.19	22.48		1.03	24.49		−1.02	37.70			
	Post	0.62	19.52	−0.09	6.56	34.80	−0.18	−16.41	37.54	0.41	−0.21	0.57
Social Anxiety Composite	Pre	0.30	0.77		0.48	0.73		0.43	0.80			
	Post	−0.15	0.94	0.52	−0.21	0.79	0.91	−0.02	0.91	0.53	0.07	−0.14
	FU	−0.26	0.99	0.63	−0.53	0.90	1.24	−0.21	0.86	0.77	0.29	−0.06
Liebowitz Social Anxiety Scale – self rated version	Pre	73.16	20.24		76.00	22.08		74.65	24.11			
	Post	57.62	24.09	0.70	56.66	24.03	0.84	62.08	25.32	0.51	0.04	−0.18
	FU	56.61	26.92	0.69	48.22	22.38	1.25	57.75	24.10	0.70	0.34	−0.04
Social Phobia Scale	Pre	37.19	14.19		38.26	13.87		38.53	13.29			
	Post	31.55	16.78	0.36	28.80	12.33	0.72	32.50	15.38	0.42	0.19	−0.06
	FU	28.86	16.44	0.54	24.00	13.78	1.03	28.25	14.42	0.74	0.32	0.04
Social Interaction Anxiety Scale	Pre	47.60	13.51		53.37	12.41		51.49	13.66			
	Post	42.12	15.77	0.37	42.66	14.42	0.80	44.43	14.91	0.49	−0.04	−0.15
	FU	40.47	16.38	0.48	37.78	17.81	1.02	42.83	14.57	0.61	0.16	−0.15
Depression (MADRS-S)	Pre	15.88	7.99		14.67	7.27		14.70	7.19			
	Post	12.14	7.52	0.48	9.66	6.30	0.74	11.03	6.53	0.53	0.36	0.16
	FU	14.14	8.32	0.21	11.32	8.74	0.42	12.14	8.21	0.33	0.33	0.24
Beck Anxiety Inventory	Pre	19.49	9.28		17.07	7.27		18.58	9.16			
	Post	13.21	7.19	0.76	10.59	5.39	1.01	15.33	10.03	0.34	0.41	−0.24
	FU	13.97	8.88	0.61	10.05	7.27	0.96	12.83	8.23	0.66	0.48	0.13
Quality of life	Pre	0.90	1.67		0.84	1.74		0.70	1.77			
	Post	1.46	1.80	0.32	1.65	1.55	0.49	0.94	1.67	0.14	0.11	−0.30
	FU	1.04	1.77	0.08	1.12	1.64	0.16	0.82	1.98	0.06	0.05	−0.12

### Predictors and moderators of change in social anxiety

#### Stimulus condition

The mixed model analysis revealed that training procedures using words were not superior to training procedures using words *and* faces in the reduction of social anxiety from pre- to follow-up-assessment (time × stimulus condition (*χ^2^*(2)  = 1.87, *p* = .39). Stimulus condition had also no differential impact on social anxiety change in the three training conditions (time × stimulus condition × training condition (*χ^2^*(6)  = 5.48, *p* = .48).

#### Fear provocation

Mixed model analyses indicated that the frequency with which participants conducted fear-provoking exercises prior to each training session did not predict change in social anxiety, as there was no significant interaction effect of time × fear provocation (*χ^2^*(2)  = 2.72, *p* = .26). Nor did it moderate the degree to which the training conditions differentially impacted on social anxiety, as the time × fear provocation × training condition interaction was not significant (*χ^2^*(6)  = 3.11, *p* = .80).

#### Pre-training bias score

In order to analyse the moderating effect of initial attention bias on change in social anxiety, we categorized participants into three groups according to their attention bias to threat index at pre-assessment. To this end, we calculated a cut-off score that reflected a meaningful difference from zero. In order to define a meaningful difference from zero, we applied the equation of the one-sample t-test: 
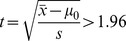
. We solved for 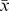 with 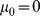
and s =  sample SD of attention bias score at pre-assessment and used 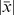 as cut-off score. Participants with attention bias to threat indices below –4.97 ms were classified as displaying an initial attention bias *away* from threat (*N* = 59) whereas participants with scores greater than 4.97 ms were categorized as displaying an initial attention bias *towards* threat (*N* = 46). Participants with scores between −4.97 ms and 4.97 ms were classified as having no initial attention bias (*N* = 24). Results of the mixed model analysis indicated that initial attention bias did not predict the magnitude of the social anxiety change over time when collapsing across training, as there was no significant interaction effect of time × initial attention bias (*χ^2^*(4)  = 3.51, *p* = .48). Neither did it moderate the differential impact of training condition on social anxiety, as the time × initial attention bias × training condition also was not significant (*χ^2^*(13)  = 8.59, *p* = .74).

### Attention bias assessment

#### Attention bias to threat at pre-assessment

To examine whether participants on average displayed an attention bias at pre-assessment, we ran three separate t-tests comparing the pre-training bias scores in each training condition to zero. Results showed that contrary to predictions for SAD participants of all three conditions did not, on average, exhibit an attention bias. Mean bias scores did not differ significantly from zero in the control condition (*t*(42)  = −.35 *p* = .73), nor in the ‘attend to threat’ condition (*t*(42)  = .28 *p* = .78), and nor in the ‘attend to positive’ condition (*t*(42)  = −.18 *p* = .86). An ANOVA showed that the three conditions did not differ in attention bias to threat indices at pre-assessment (*F*(2,126)  = .08 *p* = .93).

#### Change in attention bias

**Figure 3:**
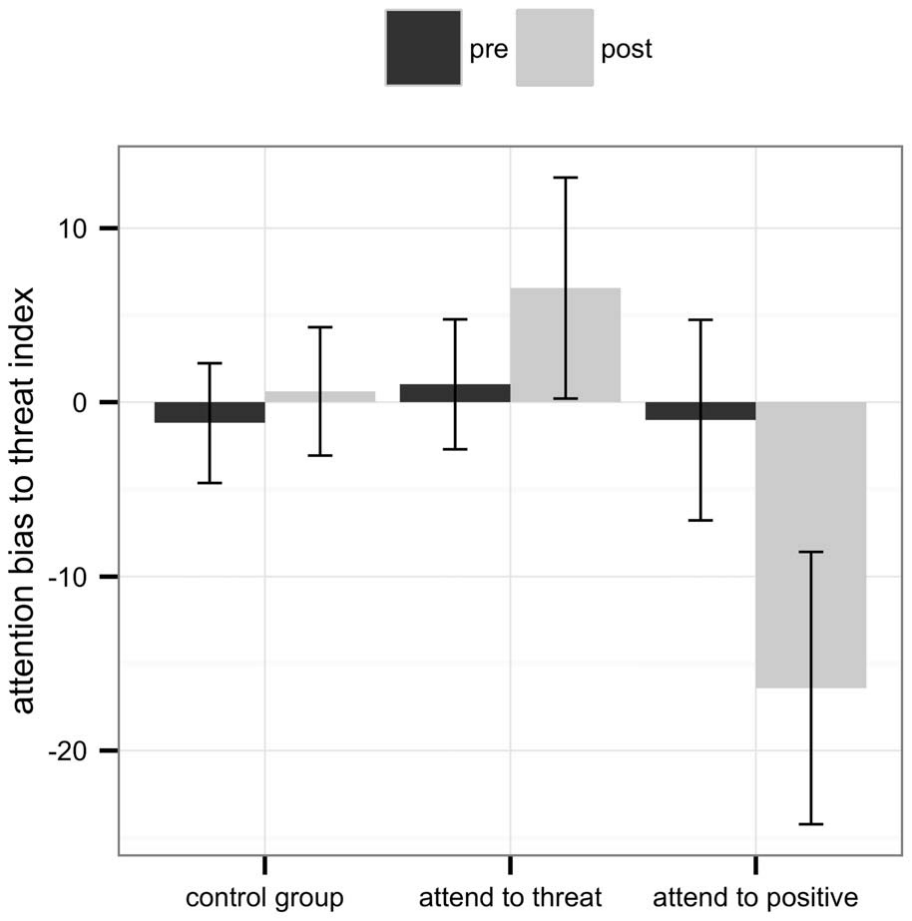
Attention bias to threat indices at pre- and at post-assessment.

Means, standard deviations and effect sizes of attention bias to threat indices are depicted in Table 2. The mixed model analysis revealed no change of attention bias from pre- to post-assessment across all three conditions (time: *χ^2^*(1)  = 0.15, *p* = .70). Across both assessment points, the training conditions did not differ in their average attention bias to threat index (training condition: *χ^2^*(2)  = 3.75, *p* = .15). Differences between the training conditions in the rate of change of attention processes did not reach significance (time × training condition: *χ^2^*(2)  = 4.48, *p* = .107). Figure 3 illustrates attention bias to threat indices in the three conditions at pre- and at post-assessment. It shows that although differences in change did not reach significance, the attention to threat indices moved in the predicted direction in both active training tasks and showed no change in the control group.

### Predictors and moderators of change in attention bias

#### Stimulus condition

Training procedures using words alone as stimuli did not lead to more change in attention bias than training procedures using words and faces as stimuli (time × stimulus condition (*χ^2^*(1)  = 2.22, *p* = .14). More importantly, the stimulus format did not affect change in attention bias differently in the three training conditions. (time × stimulus condition × training condition: *χ^2^*(4)  = 3.12, *p* = .54). The attention bias assessment included both words and faces as stimuli: The calculated attention to threat index comprises reaction times to both kinds of stimuli. To test whether the training stimuli conditions (words vs. words *and* faces) had a differential impact on bias assessed with words compared to bias assessed with faces, we repeated the mixed model analysis entering an attention to threat index based on reaction times to words alone and an attention to threat index based on reaction times to faces alone as dependent variables. Results were similar to those achieved on the general attention bias to threat index.

#### Fear provocation

Mixed model analyses indicated that the frequency with which participants conducted fear-provoking exercises prior to each training session did not predict change in attention bias, as there was no significant interaction effect of time × fear provocation (*χ^2^*(1)  = 1.48, *p* = .22). Nor did it moderate the degree to which the training conditions differentially impacted attention bias, as the time × fear provocation× training condition interaction was not significant (*χ^2^*(4)  = 1.46, *p* = .84).

#### Pre-training bias score

Mixed model analysis revealed that the initial bias score was a significant predictor of change in attention bias over time (time x initial bias: *χ^2^*(2)  = 43.73, *p*<.001). Planned comparisons revealed that, compared to individuals with no initial attention bias, change in attention bias was more pronounced in individuals that showed either an initial bias towards threat (*t*(76)  = −3.41, *p* = .001) or, as a strong statistical trend, an initial attention bias away from threat (*t*(76)  = 1.96, *p* = .054). However, this effect did not differ between the three training conditions. The initial bias did not moderate the effect of the training conditions on change in attention bias. The interaction of time x training condition x initial bias was not significant (*χ^2^*(8)  = 7.10, *p* = .53).

### Secondary outcome measures

#### Depression

Change in depression scores was analysed in a mixed model approach entering the MADRS-S score as dependent variable. In line with the analysis on social anxiety change, main effects of time and training condition as well as the interaction effect of time × condition were evaluated (see Statistical Analyses). Means, standard deviations, and effect sizes are summarized in Table 2. The analysis revealed a significant main effect of time (*χ^2^*(2)  = 42.62, *p*<.001) but no interaction effect of time × training condition (*χ^2^*(4)  = 1.85, *p* = .76). Participants in all groups showed small to moderate improvement in depression scores at post-assessment (*d* = 0.48–0.74) and a small improvement in depression scores at follow-up-assessment (*d* = 0.21–0.42), but the magnitude of these improvements did not differ between training conditions.

#### Quality of life

Potential improvements in the participants' quality of life were examined in a mixed model approach using the quality of life score as dependent variable. Again there was a significant main effect of time, indicating that across training conditions participants reported improvement in their quality of life from pre- to follow-up-assessment (*χ^2^*(2)  = 20.09, *p*<.001). There was no significant interaction effect of time × training condition (*χ^2^*(4)  = 5.12, *p* = .28), meaning that the magnitude of this improvement in quality of life did not differ between training conditions. Within group effect sizes are summarized in Table 2, from which it can be seen that this improvement in quality of life was a small effect in all training conditions both from pre- to post-assessment (*d* = 0.14–0.49), and from pre- to follow-up-assessment (*d* = 0.06–0.16).

#### Anxiety

Means, standard deviations and effect sizes for the BAI are summarized in Table 2. The mixed model analysis revealed a significant main effect of time on this measure, reflecting the fact that across training conditions participants obtained lower BAI anxiety scores at post- and follow-up-assessment than at pre-assessment (*χ^2^*(2)  = 97.85, *p*<.001). Again there was no significant time x training group interaction (*χ^2^*(4)  = 7.26, *p* = .12), meaning that the extent of this improvement in BAI anxiety scores did not differ between training conditions. Within group effect sizes in the three groups indicated small to large improvements from pre- to post-assessment (*d* = 0.34–1.01), and moderate to large improvements from pre- to follow-up-assessment (*d* = 0.61–0.96).

### Clinical change

At post-assessment, 6 (14%) participants in the control group, 9 (22%) participants in the ‘attend to threat’ group and 4 (10%) participants in the ‘attend to positive’ group were classified as improved and recovered according to the criteria suggested by Jacobson and Truax [64] (see Statistical Analyses). At follow-up-assessment, 7 (19%) participants in the CG, 11 (30%) participants in the ‘attend to threat’ group, and 5 (14%) participants in the ‘attend to positive’ group showed significant clinical change. There was no significant effect of training condition on the percentage of participants classified as improved and recovered either at post-assessment (*χ^2^*(2)  = 2.28, *p* = .31) or at follow-up-assessment (*χ^2^*(2)  = 2.84, *p* = .28).

## Discussion

The aim of the current randomised controlled trial was to examine the efficacy of different attention training conditions to change attentional processes and lead to improvements of social anxiety. The type of attention training procedure developed was novel for the current study. An ‘attend to threat’ condition was compared to an ‘attend to positive’ condition and a control condition. Differential change in social anxiety was achieved in that participants in the ‘attend to threat’ condition reported larger decreases in social fears than participants in the control condition. Critically, results on change in attention bias were not significant even though descriptive statistics showed that attention processes changed in the intended directions. Differential change in attention bias was not achieved to a sufficient degree.

A subsidiary question of the current trial comprised the direct comparison of training procedures that used words as stimuli with procedures that used words *and* faces as stimuli. A meta-analysis on attention bias modification had reported that the use of words as stimuli resulted in greater change in both attention bias and anxiety symptoms than was the case when pictorial stimuli were used [40]. In the current trial, there were no significant differences between the groups that trained with words and the groups that trained with words *and* faces. This suggests that the choice of stimulus material, at least within the current form used in this trial, does not crucially affect the efficacy of the ABM procedure for socially anxious individuals. In previous ABM studies in SAD, which all used face stimuli alone, the intended changes in attention and social anxiety have sometimes been successfully induced [33]–[37], [42], [46], and sometimes not [28], [29], [38], [39]. Clearly, something other than stimulus type must account for this inconsistency in results.

One possibility is that attention bias modification procedures might exert a greater impact on participants who already display an existing attention bias to threat as predicted by clinical models. Indeed, Amir et al. [42] demonstrated that attention bias scores moderated the efficacy of ABM in SAD. In the present study, and perhaps unexpectedly, participants did not on average display an attention bias either towards the more threatening or towards more positive stimuli prior to the training. Hence, perhaps the absence of such a pre-training attention bias in the present participants may serve to explain why the intended training conditions did not alter attention bias to a differing degree than the control condition. This notion is supported by the finding that the pre-training attention bias to threat index predicted change in attention bias and that attentional change was more pronounced in individuals exhibiting an attention bias than in individuals showing no attention bias prior to the training. However, as the subgroups in this analysis were based on the pre-scores, differences in subsequent scores could also be interpreted as regression to the mean [72]. Unfortunately, some previous studies on ABM in SAD failed to report pre-training attention bias scores [33], [34] and thus it is hard to make comparisons. In studies that support the efficacy of ABM for SAD, some have found an attention bias towards threat prior to the training [35], [36] whereas one could not detect biased attention in one of the experimental groups [42]. In studies that failed to demonstrate the efficacy of ABM, results on pre-training bias scores are also mixed. For example, Julian et al. [29] as well as Boettcher et al. [28] did not identify an attention bias towards threat prior to the training. In contrast, Neubauer et al. [39] reported that participants showed difficulties disengaging from threat. The fact that successful attention bias modification is not only, and is not necessarily, obtained using participants who show an initial attention bias, suggests that the (lack of) pre-training attention bias cannot explain all discrepancy in ABM research and that an alternative account of this discrepancy is needed.

An alternative possibility concerns the very practical issues of translating interventions from the lab to the ‘real world’, and that, for example, inconsistent findings may result from variation in delivery method. Three of the four previous studies that have failed to induce the intended differential change in attention bias and anxiety symptoms with SAD participants have delivered the programmes remotely via the internet [28], [38], [39]. In contrast, all studies that have successfully induced this attentional training effect with SAD participants have delivered the programmes within a controlled laboratory setting. This suggests that change in attention bias and symptoms may be harder to achieve using remote training delivery compared to training in the laboratory. Perhaps this reflects a particular limitation associated with remote delivery. The home setting differs from the laboratory setting in various ways. For example, individuals are more likely to feel at ease in their own home and not experience social fears potentially associated with the professional surrounding of research labs. At the same time, individuals are more likely to be distracted when training in their own home and to concentrate less on the training tasks. Given that the remote delivery of ABM programmes can modify attention bias with non-clinical samples [51], it is striking that no remotely delivered ABM has yet successfully modified attention bias with clinically anxious participants, despite the fact that laboratory-delivered ABM has been shown to do so in clinical samples of individuals with SAD, yielding very large effect sizes on social anxiety measures [33], [34]. This invites speculation that the combination of clinical dysfunction and remote delivery may exert an interactive influence that compromises the efficacy of ABM procedures. For example, clinical participants might be particularly distractible, with the consequence that the various distractions associated with the unstructured home setting disproportionately disrupt the capacity of ABM to alter attentional selectivity in clinical samples compared to non-clinical samples.

The current trial is the first study to have demonstrated that an ABM procedure designed to modify attentional response to emotional information, remotely delivered to patients with SAD, can yield a therapeutic impact on their anxiety symptoms. Unexpectedly, participants who were trained to attend to threatening cues changed more on social anxiety measures than participants in the ‘attend to positive group’ and in the control group. In this, the current results contrast most of the work on the association of attention bias towards threat and increased emotional vulnerability (see Introduction). We also note that the current pattern of results contradicts both previous trials that applied attention training towards threat. Klumpp and Amir [46] found that both active conditions, the training towards positive cues as well as the training towards negative cues, were superior to a control condition. Heeren and colleagues [35] reported that only the ‘attend to positive’ condition proved superior to a control group on behavioural and physiological measures. The methodology of the ABM procedure in the current trial differs from both previous trials in the addition of longer stimulus presentation times. To our knowledge, this is the first study that added trials with presentation times of 1000 ms. The significant impact of the ‘attend to threat’ condition could be attributed to these longer presentation times. According to the hypervigilance-avoidance model, attentional avoidance follows initial engagement with threat cues [7]. It could be argued that attentional avoidance may have been more effectively induced by the 96 trials in each session (50%) that presented stimuli for 1000 ms. However, this reasoning can only apply when the intended change in attention bias is achieved. In contrast to the two previous studies, the current trial critically did not detect clear differential change of attention bias scores across the alternative ABM conditions. In the two former studies, the ABM conditions did exert a differential impact on attentional selectivity. Hence, the change in social anxiety in these studies likely reflects the consequence of the differing changes in attentional selectivity. In the present study, given that the differential impact of the ABM conditions on social anxiety was not accompanied by a differential impact of these conditions on attention bias, the currently observed pattern of change in social anxiety may result from a mechanism other than attentional change.

What other mechanisms may account for the observed results, given that attention change itself was not detected? Given that completion of the “attend to threat” ABM condition plausibly would have involved greater attentional exposure to threat stimuli, a possible therapeutic mechanism may be simple exposure and extinction [73]. The temporal parameters of the current task could explain why it may produce greater extinction than its predecessors, as it employs longer stimulus exposure durations. If participants, during completion of the “attend to threat” ABM condition move their attention to the locus of the threat stimulus in anticipation of the probe that will appear there, then this lengthy 1000 ms stimulus presentation time will result in their greater exposure to these attended social threat stimuli. A further difference between the current approach and that adopted in other internet delivered studies was the fact that participants in all three conditions were asked to conduct small fear-provoking exercises prior to each ABM session. This instruction was designed to simulate the social anxiety and arousal level likely associated by attending training sessions in person in the laboratory (in itself an inherently social anxiety producing situation). The addition of this anxiety-induction procedure, to the systematic exposure to social anxiety-related stimuli involved in the “attend threat” condition, may further enhance the extinction resulting from this exposure. However, participants followed this instruction, on average, only on 27% of the training sessions. Furthermore, the frequency with which the instruction was followed did not moderate the degree to which the training conditions decreased social anxiety.

### Limitations

While the current study provides the important demonstration that an ‘attend to threat’ ABM procedure may attenuate social anxiety in individuals with SAD, it has limitations. First, the current study did not directly reveal the mechanism that underpinned this therapeutic effect. The findings do not clearly support the mechanism through which it was originally assumed such a procedure may alter anxiety: change in attention bias. Future studies should include comparison groups that enable to directly test alternative mechanisms of change such as exposure/extinction. Attention bias was assessed before and after exposure to the three ABM conditions. It may have been useful to have assessed attention bias and social anxiety more frequently during the training period. Not only might this have increased sensitivity to differential changes in attention bias that perhaps was less readily detected using only two assessment points, but it also could have informed whether such a change preceded and predicted change in social anxiety. Furthermore, the reliability of the applied dot-probe procedure to assess attention processes has been criticised. In several laboratory studies, the reliability of the applied attention bias assessment has been found to be poor [74]–[76]. So far, no study has evaluated its reliability when delivered remotely via the Internet. It could therefore be argued that the failure to detect differential change in attentional selectivity in the current trial merely reflects a failure to reliably assess these changes.

A second limitation is methodological and concerns the need to understand why the current and novel ABM procedure did not change bias. The current trial realised two important alterations to the standard attention modification programme: the inclusion of presentation times of 1000 ms and the instruction to conduct small fear-provoking exercises prior to the training sessions. These alterations were informed by theoretical considerations. However, as our study already included six different training groups, the present design did not effectively enable us to directly test the effects of these alterations. Furthermore, as the fear-provoking exercises were not standardised it also remains unclear what participants actually did to provoke anxiety prior to the training sessions. In contrast to some previous studies [33], [34], the current design also included positive stimuli instead of only negative and neutral stimuli and an increased number of words and faces in order to enhance the generalizability of the procedure. Future work is clearly needed that tests the results of these methodological changes in a controlled setting. Already, the sample size of the current trial restricts the interpretation of the results to the detection of moderate to large effects. Small differences in attention bias change or social anxiety change were unlikely to be detected. Meta-analyses of ABM procedures discuss the possibility of a file-drawer problem in ABM research [40], [41]. When taking into account unpublished null results studies, the real average effect of ABM on attention change might be small instead of large to moderate. In the current design, a small effect would likely not have been detected.

A third limitation is that the current emotional measures of social anxiety were based exclusively on self-report measures. No clinician-rating, behavioural measures or psychophysiological markers of social anxiety were employed. The inclusion of such measures would have allowed greater confidence that the observed beneficial effect on self-reported social anxiety generalized across these other important facets of emotional symptomatology. It is quite striking that such a simple intervention as the “attend negative” condition did substantially reduce self-reported social anxiety and convergent measure could also help tease apart the underlying mechanism.

### Conclusion and future research

Firm conclusions from ABM studies, concerning the hypothesis that attention bias change leads to a decrease of social anxiety, would require finding either a) that differential change in attention bias was induced and this was accompanied by a change in social anxiety or b) that differential change in attention bias was induced but this was not accompanied by a change in social anxiety. The former finding would support the ABM hypothesis, while the latter finding would cast doubt on its validity. When a study instead finds c) that differential change in attention bias is not induced and there is no change in social anxiety, then the status of the hypothesis is untested. Although results of types b) and of type c) both may challenge the clinical utility of the particular ABM approach under evaluation, they differ profoundly in terms of their theoretical implications, and hence also in terms of their implications for future research. If the pattern of results reveals that the target change in attention bias was successfully achieved by the intended ABM procedure, but that this attentional change did not lead to a decrease in social anxiety, then the implication would be that future research should no longer endeavour to alter attention bias in anxiety patients. In contrast, if the pattern of results instead reveals that the target change in attention bias was not achieved by the intended ABM procedure, then the implication is that future research should seek to identify better ways of achieving this attention bias change [77], [78].

Intriguingly, the results of the present study extend previous research by demonstrating a pattern of findings different from a), b) or c) as defined above. Specifically, we have shown that although the three ABM conditions did not sufficiently induce differential change in attention bias, they nevertheless did differentially influence social anxiety symptoms. Future research should determine the reliability of this finding which naturally suggests that the capacity of our procedure to reduce social anxiety need not depend upon its capacity to alter attentional bias per se but may create effects due to another mechanism entirely. As noted, the efficacy of the ‘attend to threat’ condition in reducing social anxiety may reflect the enhancement of exposure/extinction effects, resulting from exposing participants to threat related information in a manner that encouraged increased attention to this information. Hence, even if the accomplishment of attention bias modification in individuals with SAD continues to prove difficult using remote delivery of these ABM tasks, it nevertheless seems possible that therapeutic benefits may be obtained. The direct comparison of remote and laboratory delivery of attention modification tasks within a randomised controlled trial would enhance our understanding of the potential of ABM in SAD. An interest in clinical treatment development surely compels us to further understand hypothesised as well as serendipitous findings which contribute to our improved understanding of treatment gains.

## Supporting Information

Checklist S1 **CONSORT Checklist.**
(DOCX)

Protocol S1 **Trial Protocol.**
(DOC)
