# Supplementary material for: Internet-Based Attention Bias Modification for Social Anxiety: A Randomised Controlled Comparison of Training towards Negative and Training Towards Positive Cues
Source: PLoS One. 2013 Sep 30;8(9):e71760. doi: 10.1371/journal.pone.0071760 (PMC3787061; doi:10.1371/journal.pone.0071760)
Supplement: Protocol S1 — Trial Protocol. (DOC) [file pone.0071760.s002.doc]

# Study protocol

# SOFIE-11: Reduction of social anxiety through attention training via the Internet.

Social anxiety is a universal phenomenon that most individuals have experienced at some time during their lives (Kåver, 1999; den Boer, 2000). It is characterized primarily by discomfort in social and / or performance situations and a fear of being criticized or embarrassed (Furmark, Holmstrom, Savings Than, Carlbring, Andersson, 2006). Some researchers consider social anxiety as a continuum ranging from mild shyness to social phobia with its more pronounced form of generalized social phobia. According to this view, avoidant personality disorder is an extreme form of social anxiety (Rapee & Spence, 2004). Social phobia can be considered to differ quantitatively from normal shyness. The fear of social situations is so strong that it interferes with daily life. Affected individuals either avoid the feared situations, or endure them with intense anxiety (Furmark et al., 2006 , Rapee & Spence, 2004). However, there are also researchers who postulate that social phobia and its subgroups differ qualitatively from normal shyness(Hook & Valentine, 2002). The core problem in social phobia is a severe fear of getting the focus of attention of others as reflected in one or more social situations (Kåver, 1999). The most common anxiety provoking situations are public speaking situations (such as to give lectures or participate in group activities), informal interaction situations (for example, go to a party or make calls to individuals you do not know), "specific" interaction situations (talking to authorities or to assert one’s opinion) and situations of observation of behavior (for example eating or writing in front of others) (Furmark et al., 2006; Holt, Heimberg, Hope, Liebowitz, 1992). The fear of public speaking has proven to be the most common anxiety provoking situation (Furmark, 2002). Exposure to the above situations can result in varying degrees of anxiety, which manifests itself in a combination of physiological responses such as heart palpitations, tremor, flushing and sweating, combined with negative thoughts and feelings (Furmark et al., 2006; Kåver, 1999). Social phobia differs from panic disorder in the sense that the fear of symptoms is only a minor part of the symptomatology and the greater part is the fear to be critical viewed by others when these symptoms occur (Brunello et al., 2000).

DIAGNOSIS

Social phobia as a separate clinical diagnosis was highlighted by Marks in 1970 but did not own diagnostic status until it was included in the Diagnostic and Statistical Manual of Mental Disorders in 1980 (APA, 1980). The DSM criteria have subsequently been revised and since 1994, when the DSM-IV-TR was introduced, social phobia was classified as an anxiety disorder as one of the three existing classes of phobias (APA, 2000; Furmark et al., 2006). To meet the diagnosis of social phobia current diagnostic criteria require "a pronounced and persistent fear of one or more social situations or performance situations involving exposure to unfamiliar individuals or risk of critical scrutiny by others" (APA, 2000). Diagnostic criteria also pay particular attention to the extent to which the individual is limited and / or how much he/she is suffering from his/her symptoms (ibid.). Rapee and Spence (2004) argue that this is a distinctly subjective factor to consider in the diagnosis, although it is also related to the severity of social fears. Social anxiety disorder may vary on severity and duration, but the DSM-IV-TR also specifies a generalized (hereafter GSP = generalized social phobia) subtype and a non-generalized subtype(henceforth NGSP = non generalized social phobia) (APA, 2000). In its non-generalized form, it can be limited to one or two anxiety provoking situations while generalized social phobia means that most social situations arouse strong anxiety (Turk, Heimberg & Hope, 2001). In the latter case the additional diagnosis of avoidant personality should also be considered, which is characterized by a persistent and pervasive pattern of social inhibition, feelings of inadequacy, and hypersensitivity to negative evaluation (APA, 2000).

## SUBGROUPS AND AVOIDANT PERSONALITY DISORDER

Since social phobia was introduced as a clinical diagnosis, the existence of subgroups has been discussed and, particularly in recent years, the implications for treatment (Turk et al., 2001; Vriends, Becker, Meyer, Michael & Margraf, 2007). There have been discussions of whether the generalized form coincides with the axis II diagnosis of avoidant personality disorder as they are largely overlapping in terms of symptoms (Huppert, Strunk, Ledley, Davidson & Foa, 2008; Tillfors, Furmark, Ekselius & Fredrikson, 2002; Turk et al., 2001). The subgroups that are currently specified are non-generalized social phobia and generalized social phobia (Turk et al., 2001). The guidelines for determining whether it is the generalized form or not are not exact and the DSM-IV mentions that the generalized form encompasses "most social situations" without specifying a specific number (Turk et al., 2001). Studies have shown that the proportion of individuals with GSP who also meets the criteria for avoidant personality disorder is relatively high, with as much as 20 to 89% overlap in some studies (Huppert et al., 2008; Tillfors et al., 2002; Turk et al ., 2001). However, there is disagreement about whether it is relevant to speak of separate diagnoses. Huppert and colleagues (2008) suggest that there are some qualitative differences that indicate that such a division can be meaningful in a clinical setting, while Chambless, Fydrich and Rodebaugh (2008) postulate that avoidant personality is just a more severe variant of GSP. The same discussion applies to the qualitative differences between individuals with social phobia in regard to their symptoms, to the type of situations that cause anxiety and to how persistent the fear is (Turk et al., 2001). It is still disputed as to whether this heterogeneity should be seen as an expression of social phobia as a multidimensional phenomenon with the implication for adjustment of treatment, or just as an expression of the varying severity of symptoms (Vriends et al., 2007).

## EPIDEMIOLOGY AND DEMOGRAPHIC CHARACTERISTICS

Social anxiety disorder is reported to be the third most common mental disorder and the most common of anxiety disorder (Turk et al., 2001) with a lifetime prevalence in the Western world estimated at between 7 and 13% (Furmark, 2002). A review of Furmark (2002) estimated the point prevalence at 10% in Canada and at 15% in Sweden. Data of prevalence for the different subtypes are sadly deficient, but in a recent survey, about half of those with social phobia suffer from the generalized form (Hofmann, Heinrich & Moscovitc, 2004). In clinical populations GSP dominates (East, 2006). The reported prevalence rates of social phobia differ remarkably between and within cultural and geographical areas, a fact that Furmark (2002) attributes to methodological differences such as the thresholds used in the diagnosis, prevalence periods in question and so on. Furthermore, data suggest that the U.S. prevalence is on the rise, with higher lifetime prevalence rates in younger cohorts (Furmark, 2002; Magee, Eaton, Witt Chen, MacGonagle & Kessler, 1996). If this trend continues, there is reason to consider social phobia as a growing and urgent challenge for future health care (Turk et al., 2001). The onset age for social phobia is relatively low. Findings indicate a bimodal distribution with most patients being either younger than five years old or between 11 and 15 years old when the disorder begins (Schneier, Johnson, Hornig, Liebowitz & Weissman, 1992). The former group often consists of individuals who do not remember any time without troublesome social anxiety and they often belong to the subgroup with the generalized form (Rapee & Spence, 2004). Women have been found to be overrepresented in epidemiological studies, with a female-male ratio of 3:2 (Furmark, 2002; Furmark et al., 2006). Men, however, are overrepresented among those seeking help for their symptoms (ibid.). Social phobia is associated with difficulties both in work, relationships and normal daily functioning (den Boer, 2000). Research has shown that individuals with social phobia often remain single, and often divorce or separate(den Boer, 2000; Furmark, 2002). In addition, they often have a lower education and income level, lower socioeconomic status and poorer employment prospects (den Boer, 2000; Furmark, 2002). Those individuals who suffer from the generalized form of social phobia tend to have such difficulties to a greater extent than those with NGSP (Turk et al., 2001). Individuals with social phobia also report a very low quality of life, and that they feel hindered in many life domains (Erwin, Heimberg, Juster & Mindlin, 2002; Stein & Kean, 2000). If the disorder is not treated, it usually persists throughout the individual's lifetime (Brunello et al., 2000).

## COMORBIDITY

 Approximately 80% of all patients with social phobia in the general population also meet the diagnostic criteria for another axis I diagnosis (den Boer, 2000, Turk et al., 2001). In a study by Magee et al. (1996), it was found that specific phobia (37.6%), major depression (37.2%), alcohol abuse (23.9%) and agoraphobia (23.3%) belonged to the most common comorbid conditions, both in clinical and non-clinical populations. Other studies have found similar results, but also showed comorbidity with other anxiety and depressive disorders such as generalized anxiety disorder, obsessive compulsive disorder and dysthymia (Erwin et al., 2002; Hildago, Barnett & Davidson, 2001). In more than 70% of the cases, social phobia occurred first, and therefore may potentially predispose the person for further psychiatric conditions (den Boer, 2000; Turk et al., 2001, Hildago et al., 2001). However, social phobia has a relatively low age of onset, which makes it difficult to comment on causality (Rapee & Spence, 2004). Usually individuals with social anxiety disorder seek treatment in association with another diagnosis. This often implies that the underlying social phobia is overlooked and remains untreated (den Boer, 2000). Individuals with social anxiety disorder who have a comorbid mental disorder suffer to a greater extent from several negative consequences than those with uncomplicated social phobia (Turk et al., 2001). Increased suicide risk and a greater medical and psychiatric treatment seeking often are part of these consequences (Hildago et al., 2001; Schneier et al., 1992). According to Hildago et al. (2001), the suicide risk in individuals with uncomplicated social phobia is not higher than that of a control group without a diagnosis, but with comorbid disorders the suicide risk increases significantly.

### ETIOLOGY

The etiology of social phobia is not entirely known, but just as with other types of psychiatric conditions, it is considered to arise due to both genetic and environmental factors and their interaction (Tillfors, Furmark, Ekselius & Fredrikson, 2001 ; Kåver, 1999). According to Rapee and Spence (2004), it is unlikely that single risk factors contribute specifically to the development of social phobia. There rather exists a variety of factors and constellations of risks that are essential for an individual to develop the condition or not. Being born with a certain genetic predisposition, for example, constitutes an individual's "starting point". Subsequently, a variety of environmental factors and the time point, intensity, and duration of these factors contribute to the development of the disorder (Rapee & Spence, 2004). Similarly, protective factors can compensate for any genetic vulnerability (ibid.). Below is a description of the most common etiologic factors.

## GENETIC FACTORS

There is overwhelming support for the role genetic factors play in social anxiety disorder (Rapee & Spence, 2004; Stein, Chartier, Lizak & Jang, 2001). The majority of the genetic variance seems to consist of an increased risk for affective and anxiety states in general, only a small portion are genetic factors that are more specifically linked to social anxiety (Rapee & Spence, 2004). Twin studies have shown that there is a genetic predisposition to social anxiety with an estimated heredity between 0.4 and 0, 65 (ibid.). It is also common for one or more family members of individuals with social phobia to have the same difficulty or another anxiety disorder (Neal & Edelmann, 2003; Kåver, 1999; Tillfors, et al., 2001, Stein et al., 2001). This is particularly pronounced in the generalized subtype (Fors et al., 2001; Stein et al., 2001). What the hereditary component consists of is not entirely clear, but the temperament trait behavioral inhibition, which consists of withdrawal and an unwillingness to approach foreign objects / phenomena, seems to be inherited and occurs very early (usually during the first year of life) in individuals with social phobia and is likely to constitute one of several predisposing factors (Kåver, 1999; Stein et al., 2001; Neal & Edelmann, 2003).

## NEUROBIOLOGICAL FACTORS

 Social phobia can also be understood from a neurobiological perspective. Several studies in recent years have shown that individuals with social anxiety disorder have elevated nerve activity in certain parts of the brain when exposed to anxiety provoking situations compared to non-clinical control groups (Tillfors, 2001). For example, positron emission tomography (PET) studies measured higher activity in the temporal lobes, in the amygdala (body alarm system) and in the hippocampus and less activity in the prefrontal cortex (ibid.). These findings suggest a sensitive "anxiety alarm system" of this population that reacts more strongly to certain types of socially charged stimuli (Tillfors, 2001; Furmark et al., 2006). Some evidence also suggests that the dopaminergic and serotoninergic neurotransmitter systems are involved in the aetiology of social anxiety, although it is not yet fully understood how this involvement looks like (Tillfors, 2001; Kåver, 1999; Furmark et al., 2006).

## ENVIRONMENTAL FACTORS

Genetic and neurobiological factors alone cannot explain social phobia. Family and upbringing is highly relevant to the development of social anxiety (Kåver, 1999). Retrospective studies have shown a link between social anxiety and parental behaviors. Parenting styles such as over-protectiveness, high control or indifference negatively affect the childrens’ anxiety. This association is more pronounced in social anxiety disorder than in other anxiety disorders (Kåver, 1999; Neal & Edelmann, 2003; Rapee & Spence, 2004). It has also been noted that parents' behaviors and childrens’ responses are to some extent part of a reciprocal process, the importance of which is influenced by the child's age, sex and degree of social difficulties (Neal & Edelmann, 2003). Kåver (1999) also believes that the parents' ability to create opportunities for children to socialize with other matters. Socially withdrawn parents can affect children both in this respect and by modeling and observational learning. Other more general factors in childhood environment that are important for the development of social phobia are stressors such as divorce, parental conflict, parental mental illness or physical illness, poverty and difficult living conditions (Rapee & Spencer, 2004). Such factors, however, are also important to other forms of psychopathology and are not specific to the development of social anxiety (Rapee & Spence, 2004).

## BEHAVIORAL MODELS

One or more perceived failures in social situations may constitute a risk for triggering social fear by associative learning (Furmark et al., 2006; Kåver, 1999; Tillfors, 2000). According to the the model of associative learning, social phobia arises from situations of classical conditioning. The discomfort that first arose in one or two situations leads to anxiety and avoidance, and later generalizes to more and more situations (Furmark et al., 2006; Furmark, 2000). Avoidance of the social situations or the use of safety behaviors confirm social fears, and/or lead to negative reinforcement. A vicious circle is created in which the maladaptive behavior is maintained (Furmark, 2000). In a study by Stemberger, Turner, Beidel, and Calhoun (1995), 56% of those with NGSP and 40% of those with GSP reported that their social fears arose due to one or two traumatic events in the social context. East (1985) found similar results with 56.3% of patients reporting traumatic social events. Furmark (2000) argues that the results of other studies suggest that learning is a more gradual process. He suggests that conditioning of a particular context seems more likely than the conditioning of specific stimuli. This is validated by the fact that encoding of these different types of information likely involves different parts of the brain (amygdala and hippocampus) and takes some time to consolidate (Furmark, 2000).

## COGNITIVE MODELS

As for cognitive explanations of social phobia, Clark and Wells (1995) cognitive model is the most referenced. The model attempts to explain both how social anxiety arises and how it is maintained, in light of the fact that most individuals with the social phobia are regularly exposed to social situations (Clark & Wells, 1995). The model emphasizes the person's basic assumptions as they approach a social situation, which consist of previously accumulated life experiences, (Clark, 2001). Basic assumptions lead the person to perceive the situation as threatening and trigger negative automatic thoughts. Clark (2001) argues that a critical shift in the person's attention occurs in social situations, which causes intense self-observation, and leads to the perception of social ineptness and an overestimation of the negative consequences that could arise from a social meeting. In social situations, individuals also take an observer perspective in which their own experiences and cognition are taken for truth, and thus confirm an already inaccurate self-image (Clark, 2001). The model further describes how individuals with social phobia ruminate before and after a social meeting (called postmortem processing) and go through events in detail and assess their own performance very negatively (Furmark et al., 2006 , Clark, 2001; Hofman, 2007; Dannahy & transform, 2007). This factor is an important maintenance factor (Furmark et al., 2001; Clark, 2001). To avoid social mishaps, afflicted individuals also avoid situations and apply safety behaviors that further consolidate and maintain the social fear (Clark, 2001). A large number of studies provide support for several of the model's assumptions (eg Dannahy & transform, 2007; Mellings & Alden, 2000; Clark, 2001; Hofman, 2007; Musa & Lépine, 2000), but some criticism has also been directed at the concepts being difficult to operationalize and the causal relationships challenged to some extent.

## PHARMACOLOGICAL TREATMENT

Selective serotonin reuptake inhibitors (SSRIs) were originally developed as a treatment for depression. Since then, several randomized controlled trials confirmed that SSRI provide relief from social anxiety disorder and other anxiety disorders (Furmark, 2000; Rodebaugh, Holaway & Heimberg, 2004). Monoamine oxidase inhibitors (MAOIs), and benzodiazepines have also shown to provide some relief(Gould, Buckminster, Pollack, Otto & Yap, 1997). SSRIs are considered to be the first choice of pharmacological treatment because they have fewer side effects (Gould et al., 1997; Brunello et al., 2000). Beta-blockers, used for the treatment of e.g. hypertension and heart failure, sometimes are prescribed for social phobia to attenuate physiological responses. The effects were , however, challenged in later studies, especially in GSP (Furmark et al., 2006).

## PSYCHOLOGICAL TREATMENT

The majority of all research on psychological treatments for social phobia has focused on cognitive behavioral therapy, which is an umbrella term for treatments that include interventions based on behavioral theory and cognitive models (Turk et al., 2001). CBT is the treatment that has proven to have the best effect of the psychological treatments studied to date (Furmark et al., 2006). In the CBT-based approach common techniques include exposure, cognitive restructuring, social skills training and applied relaxation (Rodebaugh et al., 2004).

*Exposure:* Exposure exercises have proven effective for many types of anxiety disorders, also for social phobia (Turk et al., 2001). Exposure is based on learning theory and the fact that social situations become associated with discomfort, that is, become conditioned stimuli (Fedoroff & Taylor, 2002). Exposure means that the person is exposed to the feared situation and fully experiences the anxiety that arises until anxiety decreases (Rodebaugh et al., 2004). The mediating mechanism of exposure is still debated, but recent theories argue that it creates an opportunity for new learning that challenges the previously learned fear-response (ibid.). Exposure is often used in conjunction with cognitive restructuring (Turk et al., 2001).

*Cognitive restructuring*: Cognitive restructuring has been suggested to be important in the treatment of social phobia as the fear of negative evaluation is so prominent in this population and as this fear is a primarily cognitive phenomenon (Turk et al., 2001). Cognitive restructuring teaches the patient to identify negative automatic thoughts and basic assumptions and techniques by which these can be questioned (Turk et al., 2001; Kåver, 1999; Rodebaugh et al., 2004). Such techniques are, for example, Socratic questions or behavioral experiments (Turk et al., 2001). The goal is that the dysfunctional cognitions are replaced by more realistic and objective cognitions (Rodebaugh et al., 2004).

*Social skills training:* Social skills training (SST) is based on the assumption that individuals with social phobia lack social skills in social interaction, or that the anxiety that occurs in social contexts inhibits the skills that persons have (Rodebaugh et al., 2004; Overholser, 2002). SST therefore focuses on teaching and practicing social skills which is often done through a combination of modeling, behavioral rehearsal, corrective feedback and positive reinforcement (Rodebaugh et al., 2004; Overholser, 2002; Fedoroff & Taylor, 2002). The SST exposes the individual to multiple anxiety provoking situations, which makes it difficult to completely separate it from exposure (Rodebaugh, et al., 2004). Research into whether individuals with social phobia have a lack of skills for social interaction is inconsistent. Some studies suggest that there may be such a lack while other studies did not find evidence of this (Rodebaugh et al., 2004).

*Applied relaxation:* Applied relaxation (AR) is a method whereby the individual learns to quickly identify an anxiety response and learns to relax through breathing techniques, concentration and muscle relaxation (Fedoroff & Taylor, 2001; Rodebaugh et al., 2004; Overholser, 2002). The rationale behind AR is that relaxation counteracts anxiety when it occurs, and in the long term prevents the occurrence of anxiety (East, 1987). The patient must first learn to master the relaxation technique and then practice using it in anxiety provoking situations (Fedoroff & Taylor, 2001; East, 2001). Applied relaxation focuses on managing the physiological responses that occur in social anxiety disorder and other anxiety disorders, and is particularly recommended for patients with strong physiological reactions (Overholser, 2002). AR is rarely used as the only treatment for social phobia, but mostly in combination with other methods such as exposure and social skills training (Overholser, 2002).

## OVERVIEW OF TREATMENT EFFECTS

The pharmacological treatment of social anxiety disorder is a relatively new approach (Kåver, 1999; Rodebaugh et al., 2004). Meta-analyses found to that different pharmacological treatments have outcomes that are in the same magnitude as CBT or better (Fedoroff & Taylor, 2001; Rodebaugh et al., 2004; SBU, 2005) The combination of medication and cognitive behavioral therapy does not seem to give greater effects than each treatment alone but is insufficiently evaluated (Fedoroff & Taylor, 2001; Rodebaugh et al., 2004; SBU, 2005). Very few studies have made comparisons between CBT and medication (Fedoroff & Taylor, 2001; Rodebaugh et al., 2004). The effects of psychological treatments have been confirmed in a recent meta-analysis of Acarturk, Cuijpers, van Straten and de Graaf (2009), which found that the psychological treatment of social phobia is effective but possibly has more limited effects in severe cases. Some studies examining the outcome of CBT treatment for subgroups of generalized and non-generalized form have shown similar improvements in both groups, even after controlling for the initial severity of symptoms (Brown et al., 1995; Hope , Heimberg & Brusch, 1995). Erwin and colleagues (2002), however, could show that individuals with comorbid depressive disorders (but not other anxiety disorders) had a poorer prognosis in CBT-based group therapy. Even the most effective forms of treatment for social phobia have a large number of non-responders (about 1/6 in CBT treatments) (Rodebaugh et al., 2004; SBU, 2005). The mechanisms of effective treatment and whether different individuals respond differently to the different types of treatment is also something that is currently not known (Rodebaugh et al., 2004). MacLeod, Koster and Fox (2009) argue that it is warranted to develop treatment approaches that increase the accessibility for patients with anxiety disorders. Many patients with social phobia do not seek help, and of those who seek, few enter treatment (Olfson et al., 2000; Coles, Turk, Jindra, & Heimberg, 2004). By investigating alternative treatments it may be possible to render existing therapies more effective and to increase the availability of treatments (MacLeod et al., 2009).

## COMPUTERIZED AND INTERNET-BASED TREATMENT

Advances in information technology and increased use of computers and the Internet have contributed to the recent emergence of computer and Internet-based treatment modalities (Ritterband, Gonder-Frederick, Cox, West & Borowitz, 2003). It has also influenced the way we look at psychological treatments, for example, on how important we consider the interpersonal interaction in these contexts (template, Day & Greende, 2003; Greene & Iverson, 2009; Carlbring & Andersson, 2006.). Online therapy is already a fact (Mallen, Day & Green, 2003) and several randomized controlled trials have investigated the outcome of online therapy for a variety of psychiatric disorders, including social phobia (Carlbring & Andersson, 2006; Greene & Iverson, 2009). The majority of the applied treatments administered over the Internet have been based on cognitive behavioral therapy because this specific format is well suited to this medium (Greene & Iverson, 2009). Most online therapies have taken the form of bibliotherapy or self-help treatment with minimal therapist contact via email or telephone (Carlbring & Andersson, 2006). In summary, results of randomised controlled studies demonstrated good treatment results, many of which are clearly comparable to traditional forms of treatment, although there is still a need for more research in this area (Greene & Iverson, 2009; Carlbring & Andersson, 2006). Treatment that focuses on the training of cognitive functions for various psychiatric conditions in the form of, for example, working memory training (Klingberg et al., 2005), and, in fewer studies, applying attention training (MacLeod, Soong, Rutherford & Campbell, 2007), has also begun to appear in computerized or Internet administered shape. The benefits of computerized and Internet-based treatments are many (Greene & Iverson, 2009; Carlbring & Andersson, 2006; Ritterband et al., 2003). A clear advantage is the cost effectiveness of this type of treatment. In a "stepped care" approach, Internet-based treatments make it possible to provide a significantly greater proportion of patients with evidence-based care. Internet-based interventions require less therapist contact (Greene & Iverson, 2009). In a stepped care approach, Internet-based treatment is proposed as a first step and, after an evaluation, other forms of treatment can be offered to those who have not benefitted (ibid.). Another advantage is the possibility to reach out to the wider community, both in terms of availability of psychological therapy in general and for groups that otherwise would not have sought help at all (Marks, Shaw, & Parkin, 1998). Individuals with social anxiety have been shown to be a group who rarely seek help for their problems. Individuals with social anxiety also increasingly use the Internet to search for information about their problems and their treatment, and could thus take advantage of such opportunities as Internet-based interventions (Erwin, Turk , Heimberg, Fresco & Hantula, 2003). Another benefit of computerized and Internet-based treatments is the possibility for patients to benefit from treatment quickly and at times of day when the traditional psychological treatment is often not available. The time and cost to update any treatment manual is also lower than when the material is distributed in forms of books or papers (Marks et al., 1998; Carlbring & Andersson, 2006). For a treatment to be considered clinically effective, client compliance and satisfaction with treatment are very important factors (Greene & Iverson, 2009). A study by Proudfoot and colleagues (2003) has shown that patients with anxiety and depression are largely satisfied with Internet-administered cognitive-behavioral therapy and that drop-out is low, even lower than in traditional treatment. Disadvantages of this forms of treatment are the difficulties of establishing a diagnosis and to identify those individuals with more severe forms of psychiatric problems such as suicide risk (Carlbring & Andersson, 2006). One way to handle this is to use rigorous screening procedures that offer the option to exclude patients who exhibit severe depressive or suicidal problems early in the process (ibid.). Given that these risks also may arise or worsen during the course of treatment they should also be monitored on a weekly basis, for example. There should also be established procedures for dealing with such situations when they arise, for example by having the opportunity to contact the primary care provider (Maheu, 2003; Carlbring & Andersson, 2006). Other disadvantages mentioned are the lacking opportunity and/or the willingness of patients to make use of Internet-based or computerized therapy (Carlbring & Andersson, 2006). Reports from the Central Statistical Office (2006) show that 76% of the Swedish population has access to a computer at home and that more than 70% of those between 16 and 64 years use the Internet. The use is greatest in younger cohorts (16-19 years) and lower in older age categories (Swedish Institute for Transport and Communications Analysis, SIKA, 2006). Furthermore, the security and privacy aspects and differences in the hardware and software are also noteworthy potential disadvantages in this type of treatment. Carlbring and Andersson (2006) argue, however, that the available technology makes it possible to maintain both safety and ethical standards in these respects.

## INFORMATION PROCESSING AND ATTENTIONAL HYPERVIGILANCE

Much research has focused on the role of information processing in various states of anxiety and the question about a possible bias in relation to threatening information in terms of attention, interpretation and memory processes (Furmark, 2001; MacLeod et al., 2009). In particular, most models postulate that attention processes play a significant role in the aetiology and maintenance of social anxiety disorder (Schmidt, Richey, Buckner & Timpano, 2009; Bogels & Mansell, 2004). There are two main theoretical approaches in terms of attentional processes in relation to social anxiety. One of them, the increased self-observation, was described above as part of the cognitive model by Clark and Wells. The second is the theory of hypervigilance to threat that describes how individuals with social anxiety selective attend to threatening stimuli (Bogels & Mansell, 2004). A variety of models are based on the theory of hypervigilance and explain how these processes can be expressed more precisely (ibid.). One way to understand hypervigilance is that aversive stimuli are predominantly recognized and intensely processed. Due to the reduced capacity for habituation and an inability to re-evaluate the information, aversive stimuli continue to be perceived as threatening and thus also trigger anxiety (Mogg, Bradley, Miles & Dixon, 2004; Bogels & Mansell, 2004). In an attempt to deal with what is perceived as a threat, the increased attention is accompanied by a subsequent cognitive avoidance as reflected in a shift in attention. The avoidance constitutes a defence mechanism for the individual but also helps to maintain the symptoms (Heinrichs & Hofmann, 2001; Schmidt et al., 2009). Another approach to the theory of hypervigilance is that individuals with social phobia have difficulties disengage their attention from threatening information once they noticed it (Rapee & Heimberg, 1997). According to this view, afflicted individuals carefully scans the surrounding and are more inclined to pay attention to threatening social information, which they then have difficulty shifting their attention away from. This delay is at the expense of the perception of more neutral or positive cues (Rapee & Spence, 1997; Amir, in press; Schmidt et al., 2009). All hypervigilance models describe how individuals with different anxiety disorders direct their attention toward those stimuli that are relevant to their particular anxiety category (Bogels & Mansell, 2004). Individuals with social phobia thus experience external information, such as facial expressions, that can be associated with social threat, as more threatening than individuals without social phobia do. This is called the specificity hypothesis (Bogels & Mansell, 2004).

## EXPERIMENTAL PARADIGMS OF ATTENTION RESEARCH

To test the specificity hypothesis and theory of hypervigilance a large number of experimental studies has been conducted that can be classified under different paradigms (Bogels & Mansell, 2004). Two of the most common paradigm in the study of attention processes in social anxiety is the emotional Stroop test and the dot-probe task (Bogels & Mansell, 2004; Schmidt et al., 2009; MacLeod et al., 2009). The majority of studies have shown support for each hypothesis, but the results are not entirely consistent (MacLeod et al., 2009; Schmidt et al., 2009). Below are the main features of this research.

The emotional Stroop test boils down to name the color of a number of words printed in different colors. Selective attention is measured when the response times are longer for word that the individual associates with discomfort. Emotional Stroop studies have been used extensively in research on social phobia and have partly been able to support the theory of hypervigilance (Bogels & Mansell, 2004; Schmidt et al., 2009). Bogels and Mansell (2004) argue, however, that one should be cautious not to interpret the results of these studies as evidence of selective attention processes. Critically, other processes could partially explain the longer response times, such as cognitive avoidance, emotional reactions to the word, or preoccupation with themes and associations the word evokes (Bogels & Mansell, 2004; Schmidt et al. , 2009). Although to some extent contradictory, results of the Stroop paradigm provided evidence that individuals with social phobia exhibit greater emotional reactions to words with socially threatening meanings compared to neutral words (Bogels & Mansell, 2004). This is something that supports the specificity hypothesis, and possibly also the theory of a selective attention bias (Rinck, Becker, Kellermann & Roth, 2003; Lund & East, 1996; Bogels & Mansell, 2004; Andersson, Westö, Johansson & Carlbring, 2006).

MacLeod, Mathews and Tata (1986) were among the first to explore the differences in attention functions in individuals with anxiety disorders and developed a paradigm called dot-probe task. This was used to measure attention bias to threatening stimuli in patients with anxiety disorders (MacLeod et al., 1986). Dot-probe tasks involve two stimuli simultaneously presented on a computer screen, one containing threatening information, and one neutral stimulus (Bogels & Mansell, 2004; Schmidt et al., 2009, Amir, in press). Both stimuli are shown briefly and immediately afterwards, a letter or a character (a probe) appears behind one of them. This probe needs to be identified by the participant and indicated by pressing a corresponding button on the keyboard (Schmidt et al., 2009, Amir, in press; Bogels & Mansell, 2004). Attention bias is assessed by measuring the response times to probes behind the aversive stimuli and response times to probes behind neutral stimuli (Bogels & Mansell, 2004). The original studies generally used lexical stimuli (words) (MacLeod et al., 1986; Mathews & MacLeod, 1988). In later studies in social phobia, facial expressions that signal social threat (e.g. disgust) and neutral facial expressions were used (e.g. Schmidt et al., 2009; Amir, in press). Faces that express disgust have been chosen because they have proven to be particularly aversive for this population (Pishyar, Harris & Menzies, 2004; Amir, Najmi, Bomyea & Burns, in press). The dot-probe paradigm has several advantages over the Stroop paradigm (Bogels and Mansell, 2004). Both classes of stimuli (threatening and neutral) appear simultaneously which refines the assessment of selective attention (ibid.). Furthermore, the paradigm shows both hypervigilance and avoidance, and by its approach exclude other causes of effects, which raised criticism of the Stroop paradigm (Bogels & Mansell, 2004). At the same time, the dot-probe task and its different designs revealed some contradictory results. In a review by Mogg and Bradley (2005) based on ten articles that used the probe-detection task, the majority of studies supported the theory of selective attention in relation to threatening stimuli for individuals with generalized anxiety disorder (GAD). In a meta-analysis of Bar-Haim, Lamy, Pergamin, Bakermans-Kranenburg & van Ijzendoorn (2007), the same conclusion was drawn from studies with 172 participants with clinical anxiety or with elevated anxiety levels in comparison with control groups. The dot-probe paradigm has thus been shown to be able to measure and demonstrate attention bias.

## CONFLICTING FACTS AND EXPLANATORY MODELS

As for the role of attention in social anxiety disorder there are apparently both conflicting theories and facts. Overall, there is convincing support for individuals with social phobia to pay greater attention to threatening social information (Mogg & Bradley, 2005; Mogg et al., 2004; Pishyar et al., 2004 ; Musa & Lépine, 2000). For social phobia, facial expressions that signal anger and disgust evoke particularly strong reactions, which supports the specificity hypothesis (eg Amir, in press; Pishyar et al., 2004; Schmidt et al., 2009; Bogels & Mansell, 2004 ). Furthermore, some studies have shown that individuals with social phobia tend to avoid threatening information (Chen, Ehlers, Clark, & Mansell, 2002), while others suggested that social anxiety disorder primarily involves difficulties to disengage attention from threatening stimuli (Amir, Elias, Klumpp & Przeworski, 2003; Fox, Russo, Bowles, & Dutton, 2001). Conflicting results regarding the manner in which attention differs in individuals with social phobia show the complexity of the processes of attention and point to multifactorial causes that affect the emotional information processes (Weierich, Treat & Hollingworth, 2008). Conflicting results in different studies may also be explained in different ways. One explanation may constitute differences in how attention was measured. In some paradigms, such as the dot-probe, for example, response time is measured to detect a stimulus where both stimulus classes are presented simultaneously (MacLeod, 1986). In other paradigms, only one stimulus is presented at a time and therefore does not compete with others. Another possible explanation is that information processing, including attention bias, consists of various stages reaching from early automated responses to more conscious cognitive strategies (such as avoidance) (Mogg et al., 2004, de Ruiter & Brosschot, 1994). This is in line with the hypervigilance-avoidance hypothesis described above (Mogg, Bradley, Bono, & Painter, 1997; Andersson, et al., 2006). Shorter exposure times would, according to this model, get results showing hypervigilance while longer exposure times allow more cognitive avoidance strategies to be expressed (Mogg et al., 2004). This hypothesis has so far been shown in numerous studies, and the exposure times shown to demonstrate hypervigilance have been about 500 milliseconds while the longer exposure times (up to 1500 milliseconds) resulted in cognitive avoidance or inhibition of information (Mogg et al., 2004; Amir, Foa & Coles, 1998; Mogg & Bradley, 1999).

## ATTENTION TRAINING

The theory of hypervigilance and several cognitive models of social phobia, as previously mentioned, assume that cognitive biases maintain or affect the symptoms of the condition in a negative way (Rapee & Heimberg, 1997; Craske and Pontillo, 2001). This has also led to the hypothesis that a modification of these processes could possibly improve or abolish symptoms (Schmidt et al., 2009; Rapee & Heimberg, 1997; Craske and Pontillo, 2001). Improvements in symptoms as a result of cognitive behavioral therapy for anxiety disorders correlate with decreased attention bias in relation to threatening stimuli (Mathews, Mogg, Kentish & Eysench, 1995). This is also something that substantiated the hypothesis of a possible causal relationship of these processes (Schmidt et al., 2009). It could be speculated that the development of a training program that modifies the attention in the intended direction reduces symptoms (MacLeod, Rutherford, Campbell, Ebsworthy & Holker, 2002; Schmidt et al., 2009; Amir, in press). Numerous studies have been conducted where the aim was to test the above hypothesis and to train and modify biased attention (Amir et al., 2009; MacLeod et al., 2002; MacLeod et al., 2009; Schmidt et al., 2009). The methods have been different but in the majority of cases, a modified version of the dot-probe task was used (MacLeod et al., 2009). As was previously explained, the dot-probe task was originally used to measure attention bias. By changing the task so that the probe consistently follows one type of stimuli (e.g. neutral) attention processes are indirectly controlled and directed in the desired direction (MacLeod et al., 2009). Some of the first to experimentally explore the effect on attention processes were MacLeod and colleagues (2002). They demonstrated that dot-probe task with word stimuli could be used to modify bias attention both to and from threatening information. The participants consisted of a non-clinical population with medium anxiety levels (ibid.). The group whose attention was modified towards threatening cues exhibited higher levels of negative symptoms when exposed to a stressful situation (ibid.). This study currently provides the strongest support for the theory that individual differences in attention bias affect the sensitivity and the ability to handle stress, and increase the risk of negative affect in stressful situations (MacLeod et al., 2002; Amir et al., 2009). Based on these results, the research team thought it possible that changes in attention could be useful for treating patients with anxiety psychopathology (MacLeod et al., 2009). Numerous studies with different designs and treatment approaches have since been carried out to extend and strengthen these early findings, mostly with mainly positive or promising results (eg See, MacLeod & Bridle, 2009; Amir, in press; Amir et al., 2009 , Schmidt et al., 2009; MacLeod et al., 2007). As part of this, MacLeod et al. (2007) developed an attention training program based on the dot probe task that both measures and modifies attention bias and can be administered over the Internet. The program used neutral and aversive words as stimuli, and it was validated by two studies: one with participants with generalized anxiety disorder (GAD) and the other with volunteer psychology students. The studies confirmed the expectations that individuals with anxiety disorders had an attention bias towards threatening stimuli, while the control group avoided such words. MacLeod et al. (2007) could also show that the training program had the capacity to modify attention bias in psychology students with pre-existing bias for aversive stimuli, although changes in symptoms or anxiety levels were not measured. Schmidt et al. (2009) conducted a randomized controlled double-blind study in an attempt to further develop the method used by MacLeod et al. (2002) and investigated the effect of attention training on symptoms in a clinical population. A total of 36 individuals with generalized social phobia were randomly assigned to two groups, one took part in a modified dot-probe task, and the other group completed a very similar placebo program. Treatment was conducted in the laboratory and tasks were performed twice weekly for four weeks. Photographs of faces with different facial expressions (disgust and neutral) were used as stimuli (ibid.). To evaluate the change in symptoms both self-assessment and clinician-rated outcome measures were used. Schmidt et al. (2009) found that the group whose attention was trained away from aversive stimuli significantly improved the symptoms of anxiety and depression. A large proportion of 72% no longer met criteria for social phobia after treatment compared to 11% in the control group. Moreover, the results were stable in a follow-up after 4 months (Schmidt et al., 2009). Amir and colleagues conducted two similar studies with individuals who met criteria for GAD and individuals with generalized social phobia (GSP) (Amir et al., 2009; Amir, in press). The aim was to investigate the hypothesis that selective attention has a causal role in anxiety states and to evaluate the effects of attention training on symptoms for these groups. Amir et al. (2009) and Amir (in press) had the same study design and approach as Schmidt et al. (2009). The studies were conducted in a randomized double-blind study with a treatment and a placebo group. The number of participants totaled 29 (GAD) and 44 (GSP) who were randomized to the two groups. The treatment was performed in a laboratory environment where participants trained in total 8 times in four weeks. In the GAD study words were used as stimuli, while those who had social phobia received stimuli in the form of pictures of faces with neutral and aversive facial expressions (Amir et al., 2009; Amir, in press). Outcome measures consisted of clinician estimates, self-assessment questionnaire, and an independent measure of attention bias before and after treatment (Amir, in press; Amir et al., 2009). In both studies lower anxiety levels and a positive change in attention bias were measured in the treatment group compared to the control group after treatment (Amir et al., 2009). 50% of the participants in both treatment groups no longer met criteria for GAD and GSP after treatment. This was compared to 13% (GAD) and 14% (GSP) in the control groups (ibid.). These studies also showed stable results at 4-month follow-up.

## DIFFERENCES BETWEEN THE STUDIES

Studies conducted on programs based on the modified dot-probe task differ in several ways. A significant difference lies in the temporal scope of the program and the time frame in which participants used the program. Everything from a single training session (Amir, Weber, Beard, Bomyea & Taylor, 2008) to a day in two weeks (See et al., 2009) or two times a week for four weeks appeared (Amir, in press; Schmidt et al., 2009). Large differences also exist in in outcome measures including different forms of anxiety estimates, clinician estimates, results of social stress tasks, measures of attention bias, or a combination of these (Amir, in press; MacLeod et al., 2009). Furthermore, different types of stimuli (pictures or words) were used and the training environment varied from a laboratory environment to the home environment (MacLeod et al., 2009; See et al., 2009; Amir, in press). A few studies have also delivered attention modification programs on the Internet (MacLeod, 2007; See et al., 2009). Programs based on the modified dot-probe task have been studied both in clinical and non-clinical populations (Amir et al., 2009; MacLeod et al., 2009). In studies conducted within clinical populations, anxiety and depression were mainly investigated but studies have also been conducted in patients with eating disorders, alcohol abuse, drug abuse, and aggression problems (MacLeod et al., 2009). Despite such different study groups, differences in symptoms, and differences in methodologically, the results show a promising potential of attention training for therapeutic purposes (MacLeod et al., 2009). In summary, research on the impact of cognitive processes on various mental disorders is still in its infancy and all the evolving evidence does not necessarily prove a functional effect on symptoms of anxiety (MacLeod et al., 2009). Replication studies are needed. Studies in clinical populations can possibly contribute to the understanding of the elements of selective processing that are associated with different types of psychopathology.

## ATTENTION TRAINING

The participation in training programs intended to train attention has shown good results in previous studies (eg Amir, in press; Schmidt et al., 2009). The intended training program will be programmed in Flash to be administered over the Internet and will be identical in all important aspects to the training program used in previous studies (Amir, in press). The Amir and Schmidt's studies training program began with a short instruction and then you could start a training session, which consisted of 160 trials. As shown in the two figures below, each trial began with the display of a plus sign which was presented in the center of the screen for 500 ms. Its function was to prepare and focus participants' attention. Then two faces of the same individual appeared, one on top of the screen and one at the bottom. Each pair of stimuli showed one of two possible combinations of facial expressions, neutral-neutral or neutral-disgust. The faces of the latter combination could have two possible display modes (top or bottom of the screen). After the facial pairs were shown for 500 ms, a letter (E or F) was presented in the same location of one of faces on the screen (top or bottom). Participants were instructed to press the right arrow key if the letter that appeared was an E and the left arrow key if the letter was an F. The letter remained until a response was registered, then the next trial started with a new plus sign. Participants were instructed to work as fast as they could without sacrificing accuracy. Previous research has shown that the percentage of correct responses in this type of task is about 95% and that the time to register a response usually hovers around 600 ms after some practice (Amir, in press). The total training time for a session was about ten minutes. Each training session included a total of 160 trials, which consisted of various combinations of letters (E or F), positions (top or bottom), facial expressions (neutral or disgust) and subjects (four female and four male faces). The images were 210x158 pixels in size and appeared centered at 58 pixel spacing. Of the 160 trials, 128 (80%) consisted of neutral-disgust facial expressions and the letter replaced the neutral face in all of these cases (2 (disgust-face position: top or bottom) X 2 (letter: E or F) X 8 (individuals) X 4 (repeat)). Through this approach the participant's attention was continuously directed away from the negative stimulus, even if participants were not expressly asked to do so. This is because the letter position was predicted by the disgust-face position in 80% of the trials. The remaining 32 trials (20% of the total) consisted of the combination of neutral-neutral facial expressions and the letter positions were randomized in these cases.


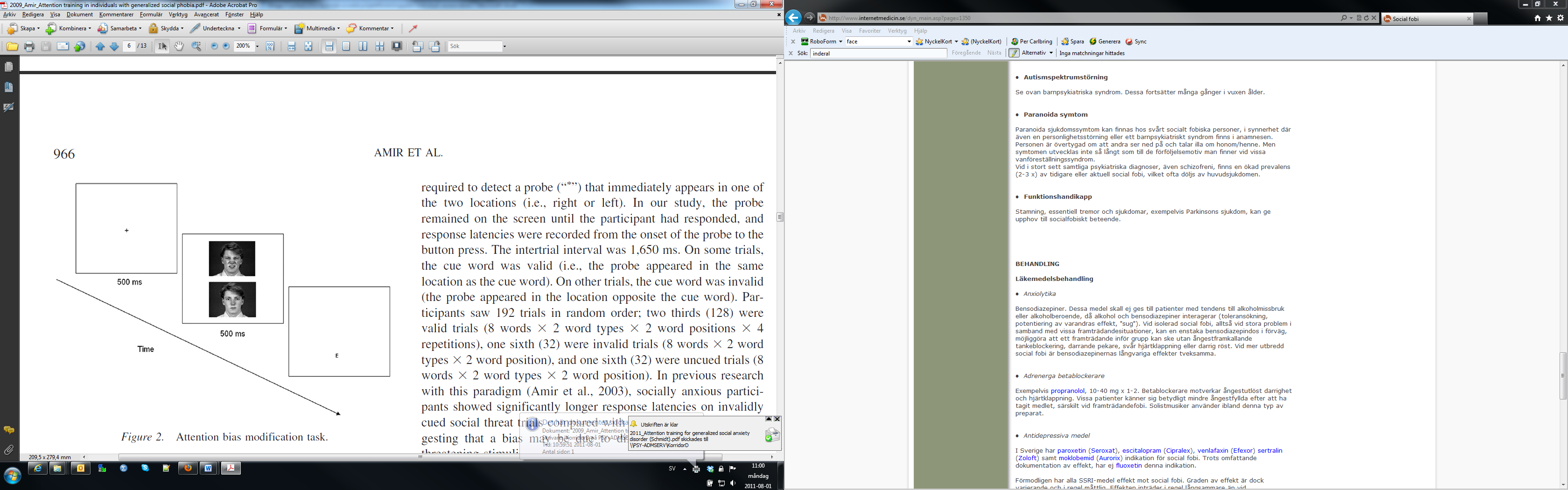


## TRAINING - PLACEBO

The placebo program’s structure was identical to the attention training program, with the difference that the letter was presented with equal frequency in the location of neutral and disgust facial expressions in the trials that involved neutral-threat presentation. In a total of 128 such trials, the letter hence followed the neutral face in 64 trials (40%), and the face that signaled disgust in 64 trials (40%). The remaining 32 trials (20% of the total) also consisted of neutral-neutral facial expressions and the letter was presented randomly equally on top or at the bottom.


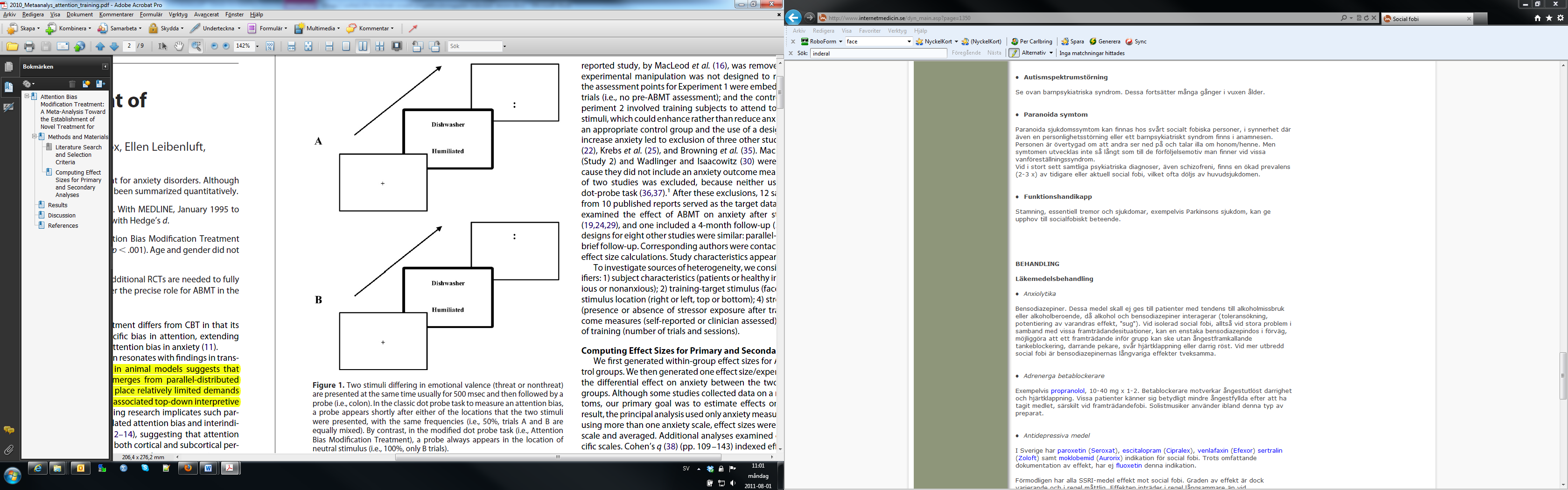


## STIMULI FOR THE MODIFICATION OF ATTENTION BIAS

The faces that appear in the program are selected from a standardized set of emotional facial expressions (Matsumoto & Ekman, 1989). From these, facial expressions of eight subjects (four men and four women) have been selected as threatening and neutral stimuli. Facial expressions that signal disgust have been selected as threatening stimuli as previous research has shown that it is associated with aversion and rejection, something this population is especially sensitive to (Pishyar et al., 2004; Amir, Najmi et al., In press). Research has also shown that individuals with social phobia have an attentional bias in relation to facial expressions that signal disgust and have difficulty shifting their attention away from such stimuli (ibid.). Finally, previous studies with the attention training program made use of these stimuli and produced a positive impact on both the aforementioned attention bias and on symptoms (Amir, in press; Schmidt et al., 2009). However, there are studies that used words instead of faces. The words have then been specific to the targeted problem. This has for example been realized in generalized anxiety disorder (GAD) with good results. It has not yet been tested in social phobia. Examples of negative words in social anxiety disorder are "judged" and "shy". They are displayed together with a neutral or positive word on the screen. The aim is that the computer program assists the participant in drawing attention away from the negative word and towards the positive word.

## Purpose

In two studies we want to examine the most effective ways to train attentional bias. That is, if the use of words, images or a combination of both affects the efficacy of attention trainings. Furthermore, we will vary the exposure time (about 400-600 ms). The purpose of the study is trying to replicate, or improve, the very good results of two previous studies (see Amir and Schmidt) where a majority of participants no longer had the diagnosis after a short training period. The main difference between our and previous U.S. studies is that we want to deliver the training program via the Internet so that participants can practice at home instead of getting to a clinic.

Research questions

The questions are: is it possible to reduce social anxiety by modifying attention with an internet based training program administered in the home? Does this treatment provide statistical and clinically significant improvements in individuals with social phobia? When does improvement occur? Finally, are treatment effects stable over time examined with follow-up assessment at 4, 12 and 24 months after training completion. The two different studies will provide answers to the necessary intensity and frequency of training exercises as well as to whether combinations of words and images are more beneficial.

## Procedure

### Study 1:

This study will be conducted at the Department of Psychology, Umeå University by Professor Per Carlbring. Research participants will be recruited through advertisements in the media. The study is presented on a website and interested participants can register with the principal investigator. The website also informs participants about ethical research principles, about the fact that participation is voluntary and that they may at any time terminate the ongoing treatment. Admission is restricted to persons over the age of 18.

The study will use a screening procedure identical to that in the recent treatment studies conducted in the SOFIE project number 8 and 9 (No. 2010-307-31 island, 2010-185-31 m). Similar screening procedures have also been used for another 20 approved studies at EPN in Uppsala, Lund, Linköping, Umeå and Stockholm.

When research participants will have expressed their interest, a study code (eg 1234xyzk) will be sent out giving access to the website. Participants log in anonymously with their study code and answer a number of self-assessment questionnaires (see Appendix 5). The questionnaires include the Social Phobia Screening Questionnaire (spsQ) and the Mongomery Asberg Depression Rating Scale. Participants must meet the criteria for social phobia according to DSM-IV. This is initially established by the spsQ, which has shown to have good sensitivity and specificity in comparison with structured diagnostic interviews. In addition, a telephone interview of the participants will be conducted, with questions based on the structured clinical interview (Structured Clinical Interview for DSM-IV Axis I Disorders: SCID I) regarding social phobia, depression and avoidant personality disorder. Telephone interviews will be conducted by psychology students on semester 10 in connection with their final thesis work under the supervision of the project manager (licensed psychologist and psychotherapist). Individuals with substance abuse problems, current major depression and / or suicide risk will be excluded from the study. Furthermore, the research participants submit a written signed consent form, which includes a statement on the processing of personal data in accordance with the Data Protection Act and which states that they have taken note of the information presented on the website about the study process and the conditions that apply (see Annex 4).

Applicants who meet the criteria will be randomized into two groups so that half will undergo an effective attention training (experimental group) and the other half may start a supposedly less effective attention training (control group).

Prior to the treatment all research participants fill in outcome questionnaires that include of the following: Social Interaction Anxiety Scale, Social Phobia Scale, Liebowitz Social Anxiety Scale, MADRS-S, STAI-S, STAI-T, and Quality of Life Inventory (see Appendix 5 ). All forms are internationally established and have been shown to have good psychometric properties. These questionnaires will be administered at all measurement occasions.

Via the Internet, participants will take part in an attention training program (see description above) lasting for 14 days of active treatment. During this period, the exercises will be done with an intensity of 10 minutes of exercise per day.

Outcome questionnaires are administered 24 hours after the last training session. Follow-up assessments are then conducted after 4 months, 12 months and 24 months to evaluate the long-term treatment effects, and to compare these with the short-term effects. Pre-, post-, and follow-up measurements will be made with internationally established forms (see above) and the result will be calculated and statistically analyzed and reported at the group level.

### Study 2

The study applies the same procedure as Study 1, but instead of 14 consecutive days of daily training the training period will be extended to 4 weeks of active treatment with only two training sessions a week.

## Sample size

The sample size is expected to amount to 300 persons, 75 in each group per study. A total of 300 research participants will be recruited. Based on the power calculation (80% power), 75 participants in each group will be sufficient to provide a reasonable chance to detect clinically relevant effects, and also to be identify predictors with logistic regression. The standardized mean difference in previous studies is admittedly large (approximately d = 0.80), but since we are interested in predictors a larger sample is required. This is why 150 individuals per subproject are required.

## Selection

Research participants who will be persons of age, will be recruited through the project's web-based interest notification page, and from advertisements in the media. Participants must meet the criteria for social phobia according to the DSM-IV. Individuals with such ongoing major depression and / or individuals who are considered suicidal will be excluded from the study.

## Consent

Informed consent will be obtained in connection with the screening procedure. Written informed consent will state that participants agree to the processing of personal data in accordance with the Data Protection Act, and also that he / she received information on the website about the study’s process and the conditions applied (Annex 4).

## Ethics

Research participants participate voluntarily in the study. Participants will be informed on the potential benefit of the computerized attention training. Moreover, participants will be informed that any improvement requires active engagement.

The following ethical risks have been identified: the first is that participants who need specialized medical investigations could be included. At the slightest suspicion of unclear etiology, participants will be asked to consult a doctor in the community. The other risk is that deeply depressed individuals are included. Suicidal individuals will be excluded. This is done through a combination of the self-rating version of the Montgomery Asberg Depression Rating Scale and a clinical interview by telephone.

The individuals that are excluded from the study may perceive it negatively. All individuals who are excluded, therefore, will get advice on how and where they can seek alternative treatment in the community, and in cases where it is deemed appropriate recommendations for appropriate self-help literature will be made. Participants will be randomly allocated to one of two study groups (attention training or control condition). Best scientific practice does not allow the participants themselves to select the group and the individuals who do not agree to this will not be included in the study. Which participants belong to which group is also unknown to the research staff and disclosed only after the end of treatment at 4-month follow-up. Participants who are randomly assigned to the control group can take part in the attention training program after follow-up at 4 months. An obvious disadvantage of those randomly assigned to the control group is that they are likely to have a less effective treatment. Previous studies have shown positive effects in the control group, but more modest than in the experimental group. For this reason, all participants will be offered the supposedly more effective treatment after 4 months. It should be added, however, that the effects of being in the control group seem significantly larger than the expected spontaneous improvement in the same population.

## Publication

The results will be published in international peer-review journals. Where possible, the results will be published in journals with open access.
